# Exploring Kinase Inhibition Properties of 9*H*-pyrimido[5,4-*b*]- and [4,5-*b*]indol-4-amine Derivatives

**DOI:** 10.3390/ph13050089

**Published:** 2020-05-09

**Authors:** Yvonnick Loidreau, Carole Dubouilh-Benard, Marie-Renée Nourrisson, Nadège Loaëc, Laurent Meijer, Thierry Besson, Pascal Marchand

**Affiliations:** 1 Normandie Université, UNIROUEN, INSA Rouen, CNRS, COBRA UMR 6014, F-76000 Rouen, France; yvonnick.loidreau@gmail.com (Y.L.); carole.dubouilh@univ-rouen.fr (C.D.-B.); 2 Université de Nantes, Cibles et Médicaments des Infections et du Cancer, IICiMed, EA 1155, F-44000 Nantes, France; marie-renee.nourrisson@univ-nantes.fr; 3 Station Biologique de Roscoff, Protein Phosphorylation & Human Disease Group, 29680 Roscoff, France; nadege.loaec@univ-brest.fr (N.L.); meijer@perha-pharma.com (L.M.); 4 Perha Pharmaceuticals, Perharidy Peninsula, 29680 Roscoff, France

**Keywords:** microwave-assisted chemistry, protein kinases, CK1, DYRK1A, CDK5, GSK-3

## Abstract

We previously highlighted the interest in 6,5,6-fused tricyclic analogues of 4-aminoquinazolines as kinase inhibitors in the micromolar to the nanomolar range of IC_50_ values. For the generation of chemical libraries, the formamide-mediated cyclization of the cyanoamidine precursors was carried out under microwave irradiation in an eco-friendly approach. In order to explore more in-depth the pharmacological interest in such tricyclic skeletons, the central five member ring, i.e., thiophène or furan, was replaced by a pyrrole to afford 9*H*-pyrimido[5,4-*b*]- and [4,5-*b*]indol-4-amine derivatives inspired from harmine. The inhibitory potency of the final products was determined against four protein kinases (CDK5/p25, CK1δ/ε, GSK3α/β, and DYRK1A). As a result, we have identified promising compounds targeting CK1δ/ε and DYRK1A and displaying micromolar and submicromolar IC_50_ values.

## 1. Introduction

**Figure 1:**
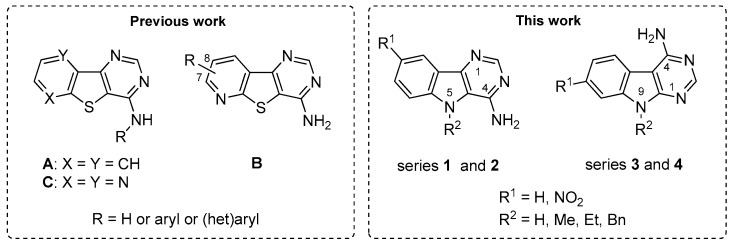
Previous benzo[*b*]thieno[3,2-*d*]pyrimidin-4-amines (**A**) and their pyrido (**B**) and pyrazino (**C**) analogues (**left**). The 9*H*-pyrimido[5,4-*b*]indol-4-amine derivatives (**1** and **2**) and their 9*H*pyrimido[4,5-*b*]indole isomers (**3** and **4**) described in this work (**right**).

Protein kinases are an important family of enzymes able to phosphorylate tyrosine (Tyr) and serine (Ser)/threonine (Thr) residues present in various proteins [1]. Abnormal protein kinase regulation and phosphorylation are now associated with numerous diseases including cancer [2,3], and neurodegenerative disorders [4,5,6]. In the last decade, about 300 protein kinase inhibitors were involved in clinical trials and 49 have been recently approved by the US Food and Drug Administration (FDA), mostly tyrosine kinase inhibitors, and mainly for cancer therapy [7]. In the same period, our groups have been particularly invested in the development of efficient and eco-compatible chemical methodologies allowing rapid access to libraries of potent bioactive arenes and their heteroarenes analogues. Studying ancestral thermal-sensitive reactions for which usual methods require forcing conditions or prolonged reaction times (e.g., Niementowski reaction [8,9] and Dimroth transposition [10]), microwave-assisted syntheses of novel benzo[*b*]thieno[3,2-*d*]pyrimidin-4-amines (series **A**) [11,12] and their pyrido (series **B**) and pyrazino analogues (series **C**) [13,14] have been successfully described (Figure 1). In this context, more than one hundred derivatives of the heterocyclic systems have been studied. Among them, the pyrido[*b*]thieno[3,2-*d*]pyrimidin-4-amine derivatives (series **B**) have shown the most interesting selectivity and inhibitory potency towards CK1δ/ε over the other tested enzymes (CDK5/p25, GSK3α/β, and Dyrk1A) [14].

In an effort to expand the chemical space and to highlight efficient kinase inhibitors, the synthesis of indole counterparts of the previously-described series has been envisaged. Such compounds are considered as analogues of harmine (see **11a** in Scheme 2), a natural alkaloid that still generates a lot of work in the hope of developing therapies for Alzheimer’s disease (AD) and Down syndrome (DS) [15,16]. This paper describes simple and convenient synthetic routes to 9*H*-pyrimido[5,4-*b*]indol-4-amine derivatives (series **1** and **2**) and their 9*H*pyrimido[4,5-*b*]indole isomers (series **3** and **4**). The chemistry described in this paper was mainly carried out under microwave irradiation in an eco-friendly approach. Kinase inhibition of the products obtained was evaluated on an array of four Ser/Thr kinases (CDK5/p25, CK1δ/ε, DYRK1A, and GSK3α/β), all members of the CMGC kinase family, chosen for their strong implication in various cellular regulation processes [17,18,19,20,21,22,23,24,25].

## 2. Results

### 2.1. Chemistry

Synthesis of series **1**, **2**, **3**, and **4** was inspired from our previous works on various benzo-, pyrido-, and pyrazino[*b*]thieno[3,2-*d*] pyrimidines (see series **A**, **B** and **C** in Figure 1) [11,12,13,14]. The nitrogen ring in pyridine and in pyrazine may be considered to be deactivated compared with benzene. It is sometimes compared with nitrobenzene as the molecules behave similarly in relation to the deficit of electron density. This consideration guided the choice of the nitro substitution (R^1^) on the starting indole derivatives for completion of the structure-activity relationship (SAR) study. The *N’*-(cyano-1*H*-indolyl)-*N,N*-dimethyl formimidamide precursors (**7a**–**d**, **8a**–**d**, **9a**–**d**, and **10a**–**d**) were heated at 170–200 °C under microwaves in the presence of an excess of formamide (40 equiv.) (for reaction times and yields see Table 1).

**Scheme 1:**
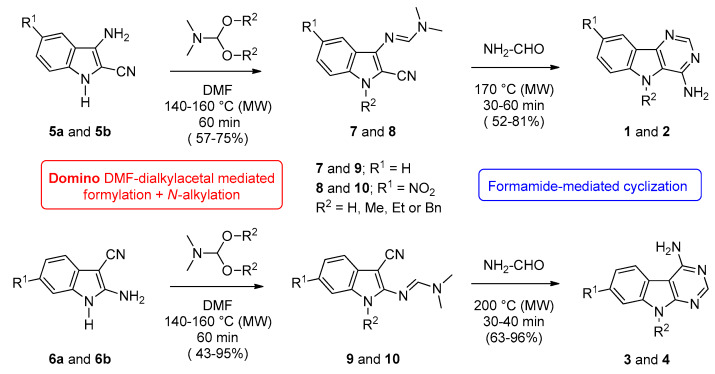
Synthesis of 9*H*-pyrimido[5,4-*b*]indol-4-amines (series **1a**–**d** and **2a**–**d**) and their 9*H-*pyrimido[4,5-*b*]indoles isomers (series **3a**–**d** and **4a**–**d**).

Functionalized indoles (**7a**–**d**, **8a**–**d**, **9a**–**d**, and **10a**–**d**) were previously obtained from 3-amino-1*H*-indole-2-carbonitriles (**5a**,**b**) or their 5-nitro-3-amino-1*H*-indole-2-carbonitrile isomers (**6a**,**b**) which were condensed with 10 equiv. of DMF-dialkylacetals like *N,N*-dimethylformamide dimethyl acetal (DMF-DMA), *N,N*-dimethylformamide diethyl acetal (DMF-DEA), and *N,N*-dimethylformamide dibenzyl acetal (DMF-DBA), respectively (Scheme 1). A strict control of the experimental conditions in terms of temperature and reaction time allowed access to the expected compounds in good to excellent yields (for details on suggested mechanism and experimental details see ref. [26]).

**Scheme 2:**
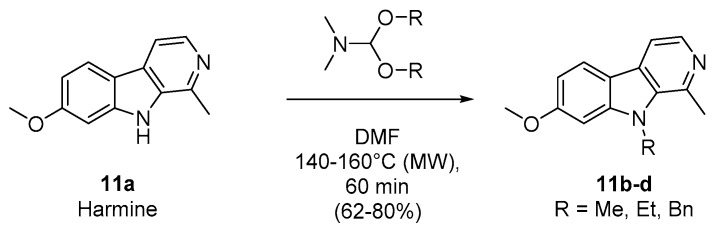
Microwave-assisted synthesis of *N*-alkylated harmine derivatives (compounds **11b**–**d**).

To compare the biological results of harmine (**11a**) and some of its *N9*-alkylated derivatives (**11b**–**d**) with the pyrimidoindoles prepared in this work (series **1**, **2**, **3**, and **4**, Table 1), we decided to explore the capacity of DMF-dialkylacetals to transfer an alkyl group to nucleophilic atoms, as an interesting alternative to previous methods [27,28,29,30]. Then, harmine was heated under controlled microwave-assisted heating in sealed vials (10 mL) in the presence of 10 equiv. of DMF-DMA, DMF-DEA, or DMF-DBA. The corresponding *N9*-alkylated 7-methoxy-1-methyl-β-carbolines (**11b**–**d**) were obtained in good yields (Scheme 2, Table 2).

### 2.2. Biological Evaluation

The inhibitory potency of the synthesized pyrimido[4,5-*b*]indol-4-amines and pyrimido[5,4-*b*]indol-4-amines towards CDK5/p25, CK1δ/ε, DYRK1A, and GSK-3α/β was investigated according preceding procedures [11,12,13,14]. Data are listed in Table 3 including results obtained with harmine (**11a**) and its congeners (**11b**–**d**).

All the tested compounds were inactive against CDK-5/p25 and GSK3α/β. 8-Nitro-5*H*-pyrimido[5,4-*b*]indol-4-amines (**2a**–**d**) were also inactive against the two other tested kinases, except compound **2a** which exhibited a micromolar range IC_50_ value (7.6 μM) against DYRK1A. General comparison of series **1**, **3**, and **4** revealed a similar inhibitory activity against the array of four kinases. Values were mainly in the micromolar range against CK1δ/ε, except compounds **1d** and **3a** which disclosed submicromolar IC_50_ values (0.6 and 0.7 μM, respectively). It can be noted that **1d** seemed more specific for CK1δ/ε in view of its lack of activity against DYRK1A, whilst **3a** maintained a micromolar affinity (IC_50_ = 3.1 μM) against this biological target.

Considering CK1δ/ε kinase inhibition, the introduction of a nitro group was deleterious for series **1** vs. series **2**, whereas inhibitory activity remained for series **3** vs. series **4**, depending of the orientation of the aminopyrimidine ring in the fused system and its relative position to the nitro group. On the other hand, for the unsubstituted tricyclic derivatives on indole (series **1** and **3**) the position of the amino group does not seem critical since micromolar CK1 inhibition was conserved within the two series.

Interestingly, a loss of DYRK1A inhibitory activity was observed for the compounds bearing a *N*-methyl or a *N*-benzyl chain compared to their NH or *N*-ethyl analogues, except for the inactive series **2** and the very active compounds **11a**–**d**.

Tested as a positive control under the same conditions as series **1**–**4**, harmine (**11a**) was definitely inactive against CDK5/p25 and GSK3α/β. This natural product shows interesting activity against DYRK1A and a weak inhibition of CK1δ/ε (IC_50_ = 1.5 μM) as previously mentioned in various papers relating the use of harmine for treatment of neurodegenerative diseases [31,32]. The *N*-methyl, *N*-ethyl, and *N*-benzyl derivatives (**11b**, **11c**, and **11d**) were totally inactive against the three kinases CDK5/p25, CK1δ/ε, and GSK3α/β. Their affinity was focused on DYRK1A with interesting IC_50_ values, quite close to the nanomolar range IC_50_ obtained for the lead harmine (**11a**) (see Table 3), confirming recently-published results [29].

## 3. Discussion

Results described above confirm the interest in developing chemical methods that allow easy access to libraries of various potentially bioactive molecules. This is particularly noticeable in the case of compounds **1a** and **1b**, which are the only derivatives of these new series that were already synthesized via multistep processes (2–4 steps), in long time reactions (6–20 h) and sometimes difficult operating conditions, using toxic reagents (e.g., POCl_3_) [33]. The combination of microwave-assisted heating and the use of DMF-dialkylacetals provided in all cases short reaction times and comfortable operating conditions.

The two unsubstituted isomeric forms (series **1a**–**d** and **3a**–**d**) expressed a similar average activity against the two kinases CK1δ/ε and DYRK1A. Moreover, the data obtained for the two series of pyrimidoindoles bearing a nitro group, demonstrate that the pyrimido[4,5-*b*]indol-4-amines (series **4a**–**d**) are more active than their pyrimido[5,4-*b*]indol-4-amine isomers (series **2a**–**d**). In case of the first series (**1a**–**d** and **2a**–**d**), the data suggest that addition of a nitro group significantly decreases the inhibitory activity. In contrast, the two sets of pyrimido[4,5-*b*]indol-4-amines (**3a**–**d** and **4a**–**d**) exhibit similar micromolar range IC_50_ values which are independent of the substitution pattern on the benzene moiety of the indole ring.

**Figure 2:**
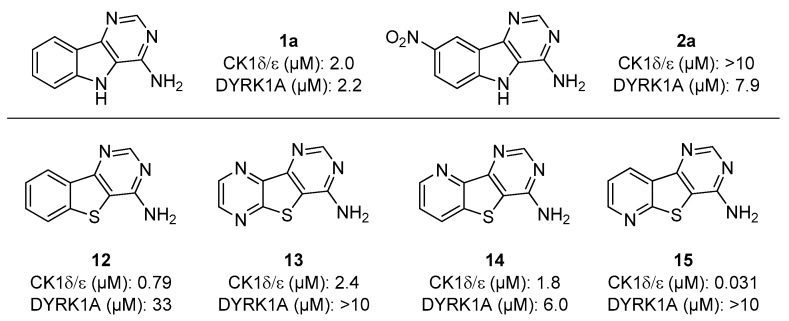
Comparison between selected pyrimido[4,5-*b* or 5,4-*b*]indol-4-amines described in this work and some of their thieno[2,3-*d*]pyrimidin-4-amine congeners (**12**, **13**, **14** and **15**) described in preceding work.

Figure 2 focuses on the two kinases mainly inhibited in this study and the IC_50_ values towards CK1δ/ε and DYRK1A protein kinases are reported for selected pyrimido[4,5-*b* or 5,4-*b*]indol-4-amines (**1a**, **2a**, **3a**, and **4a**). Inhibitory activity is compared with the biological data already published for thieno[2,3-*d*]pyrimidin-4-amine analogues described as potential Ser/Thr kinases inhibitors [12,13,14].

It can be observed that the replacement of the sulfur atom, in the structure of compound **12**, by a nitrogen atom (NH, more exactly, in compound **1a**) resulted in increasing DYRK1A inhibitory potency (from 33 to 2.2 µM) and keeping CK1 activity. The major difference between the two compounds lies in the nature of the hydrogen bonding (acceptor for S vs. donor for NH) that would allow a particular binding mode and interaction with the target protein, explaining this trend. When comparing the activity of nitroindole derivative **2a** with the deactivated analogues **13**, **14**, and **15**, due to the presence of a pyrazine or a pyridine ring, opposite selectivity towards CK1δ/ε and DYRK1A was observed. Indeed, the last ones displayed micromolar and submicromolar activity against CK1 and no activity on DYRK1A, whereas compound **2a** exhibited no inhibition against CK1δ/ε and low micromolar activity towards DYRK1A. Again, the four compounds behave identically in terms of electronic deficiency but, for three of them, we assumed that the presence of a nitrogen atom could bring an additional possibility of interaction with the protein through the lone pair of electrons.

## 4. Materials and Methods

### 4.1. General Information

All reagents were purchased from commercial suppliers and were used without further purification, except for DMF, which was stored under argon and activated molecular sieves. All reactions were monitored by thin-layer chromatography with silica gel 60 F254 (Merck KGaA, Darmstadt, Germany) precoated aluminium plates (0.25 mm). Visualization was performed with a UV light at wavelengths of 254 nm. Purifications were conducted with a flash column chromatography system equipped with a dual UV/vis spectrophotometer (200–600 nm), a fraction collector (176 tubes), a dual piston pump (1 to 200 mL/min, Pmax = 15 bar), which allowed quaternary gradients, and an additional inlet for air purge ((Puriflash, Interchim, Montluçon, France). Melting points of solid compounds were measured with a SMP3 melting point instrument (STUART, Bibby Scientific Ltd., Roissy, France) with a precision of 1.5 °C. IR spectra were recorded with a Spectrum 100 Series FTIR spectrometer (PerkinElmer, Villebon S/Yvette, France). NMR spectra (^1^H and ^13^C) were acquired at 295 K using an AVANCE 300 MHz spectrometer (Bruker, Wissembourg, France) at 300 and 75.4 MHz, using trimethylsilane (TMS) as an internal standard. Coupling constants *J* are in Hz, and chemical shifts are given in ppm. Mass spectrometry was performed by the Mass Spectrometry Laboratory of the University of Rouen. The mass spectra electrospray ionization (ESI), electron impact ionization (EI), and field desorption (FD) were recorded with an LCP 1er XR spectrometer (WATERS, Guyancourt, France). Microwave experiments were carried out at atmospheric pressure in 50–250 mL round bottom flasks fitted with a reflux condenser, in a RotoSYNTH^TM^ (Milestone S.r.l., Milano, Italy), a multi-mode cavity microwave reactor designed for synthetic chemistry (0–1200 W). Microwave reactions in sealed tubes (10 mL) were performed with a Initiator^TM^ microwave synthesis instrument (Biotage, Uppsala, Sweden) (0–400 W). Temperatures of the reactions were monitored via IR-sensors. The percentage of purity of all tested products was more than 95% determined by high pressure liquid chromatography (HPLC) analysis. ^1^H-NMR and ^13^C-NMR spectra of new compounds are available in Supplementary Materials Section Figures S1–S16.

### 4.2. Chemistry

All details conerning the synthesis of *N’*-(3-Cyano-1-alkyl-1*H*-indol-2-yl)-*N,N*-dimethylformimidamide intermediates (**7a**–**d**, **8a**–**d**, **9a**–**d**, and **10a**–**d**) are described in a preceding work [26].

Compounds **1a** and **1b** were already described and data given in the corresponding patent are only ^1^H NMR spectra [33]. Harmine derivatives **11b**, **11c**, and **11d** were described in a preceding work [28] cited in some recent studies [29,30].

#### 4.2.1. General Procedure for the Synthesis of 5*H*-Pyrimido[5,4-b]indol-4-amines (Series 1 and 2) and 5*H*-Pyrimido[4,5-b]indol-4-amines (Series 3 and 4).

Formamide (40 equiv.) was added to the formimidamide precursor (**7b**–**d**, **8b**–**d**, **9b**–**d**, or **10b**–**d**) (1 mmol) and the mixture was heated under microwave irradiation (200 W). On completion, the reaction was cooled to room temperature and water was added. The solid was filtered off, washed with water, and dried. The crude solid was purified by silica gel column chromatography using PE/EtOAc (100:0 to 0:100, *v*/*v*) as the eluent to give the desired compounds.

*5H-Pyrimido[5,4-b]indol-4-amine* (**1a**): brown powder (0.149 g, 81%) obtained from **7a** after 30 min of irradiation at 170 °C according to the general procedure; mp > 320 °C; IR (neat) ν_max_(cm^−1^): 3047, 1616, 1600, 1559, 1498, 1462, 1428, 1354, 1343, 1316, 1295, 1234, 1207, 1119, 756, 745; ^1^H NMR (300 MHz, DMSO-*d*_6_): δ 10.98 (br s, 1H, N*H*), 8.29 (s, 1H, *H*-2), 8.05 (d, 1H, *J* = 8 Hz, *H*-8), 7.63 (d, 1H, *J* = 8 Hz, *H*-6), 7.50 (td, 1H, *J_1_* = 2 Hz, *J_2_* = 8 Hz, *H*-7), 7.22 (td, 1H, *J_1_* = 2 Hz, *J_2_* = 8 Hz, *H*-6), 6.95 (s, 2H, N*H*_2_); ^13^C NMR (75 MHz, DMSO-*d*_6_): δ 150.8, 149.9, 141.9, 138.8, 127.7, 120.9, 120.4, 119.5, 117.4, 112.5; HRMS calcd for C_10_H_9_N_4_ [M + H]^+^ 185.0827 found 185.0822

*5-Methyl-5H-pyrimido[5,4-b]indol-4-amine* (**1b**): brown powder (0.115 g, 58%) obtained from **7b** after 30 min of irradiation at 170 °C according to the general procedure; mp 201–202 °C; IR (neat) ν_max_(cm^−1^): 3074, 1657, 1623, 1590, 1537, 1493, 1462, 1431, 1400, 1352, 1329, 1244, 962, 842, 789, 737; ^1^H NMR (300 MHz, DMSO-*d*_6_): δ 8.29 (s, 1H, *H*-2), 8.06 (d, 1H, *J* = 8 Hz, *H*-9), 7.69 (d, 1H, *J* = 8 Hz, *H*-6), 7.58 (td, 1H, *J_1_* = 2 Hz, *J_2_* = 8 Hz, *H*-7), 7.24 (td, 1H, *J_1_* = 2 Hz, *J_2_* = 8 Hz, *H*-8), 6.93 (s, 2H, N*H*_2_), 4.08 (s, 3H, C*H*_3_); ^13^C NMR (75 MHz, DMSO-*d*_6_): δ 151.4, 149.7, 140.9, 128.0, 120.4, 120.3, 119.5, 114.2, 110.4, 106.3, 31.7; HRMS calcd for C_11_H_11_N_4_ [M + H]^+^ 199.0984 found 199.0981.

*5-Ethyl-5H-pyrimido[5,4-b]indol-4-amine* (**1c**): brown powder (0.136 g, 64%) obtained from **7c** after 30 min of irradiation at 170 °C according to the general procedure; mp 161–162 °C; IR (neat) ν_max_(cm^−1^): 3349, 1641, 1589, 1532, 1402, 1382, 1334, 1226, 1046, 1024, 988, 827, 729; ^1^H NMR (300 MHz, DMSO-*d*_6_): δ 8.31 (s, 1H, *H*-2), 8.08 (d, 1H, *J* = 8 Hz, *H*-9), 7.72 (d, 1H, *J* = 8 Hz, *H*-6), 7.58 (td, 1H, *J_1_* = 2 Hz, *J_2_* = 8 Hz, *H*-7), 7.22 (td, 1H, *J_1_* = 2 Hz, *J_2_* = 8 Hz, *H*-8), 6.90 (s, 2H, N*H*_2_), 4.61 (q, 2H, *J* = 7 Hz, C*H*_2_), 1.21 (t, 3H, *J* = 7 Hz, C*H*_3_); ^13^C NMR (75 MHz, DMSO-*d*_6_): δ 150.9, 149.7, 140.1, 128.1, 120.7, 120.5, 119.6, 118.2, 112.1, 110.4, 39.7, 16.0; HRMS calcd for C_12_H_13_N_4_ [M + H]^+^ 213.1140 found 213.1133.

*5-Benzyl-5H-pyrimido[5,4-b]indol-4-amine* (**1d**): yellow powder (0.143 g, 52%) obtained from **7d** after 40 min of irradiation at 170 °C according to the general procedure; mp 219–220 °C; IR (neat) ν_max_(cm^−1^): 3062, 1641, 1619, 1581, 1530, 1494, 1453, 1403, 1378, 1330, 1254, 1207, 1193, 954, 754, 734; ^1^H NMR (300 MHz, DMSO-*d*_6_): δ 8.33 (s, 1H, *H*-2), 8.10 (d, 1H, *J* = 8 Hz, *H*-9), 7.79 (d, 1H, *J* = 8 Hz, *H*-6), 7.55 (td, 1H, *J_1_* = 2 Hz, *J_2_* = 8 Hz, *H*-7), 7.28-7.17 (m, 4H, *H*-8, and *H*-ar), 7.00 (dd, 2H, *J_1_* = 1 Hz, *J_2_* = 8 Hz, *H*-ar), 6.90 (s, 2H, N*H*_2_), 5.87 (s, 2H, C*H*_2_); ^13^C NMR (75 MHz, DMSO-*d*_6_): δ 151.0, 150.0, 143.8, 141.0, 138.3, 128.5 (2C), 128.3, 127.2, 126.2 (2C), 120.9, 120.5, 120.0, 118.0, 111.1, 46.9; HRMS calcd for C_17_H_15_N_4_ [M + H]^+^ 275.1297 found 275.1295.

*8-Nitro-5H-pyrimido[5,4-b]indol-4-amine* (**2a**): brown powder (0.149 g, 65%) obtained from **8a** after 60 min of irradiation at 170 °C according to the general procedure; mp > 320 °C; IR (neat) ν_max_(cm^−1^): 3102, 1692, 1627, 1597, 1548, 1512, 1474, 1453, 1377, 1321, 1303, 1233, 1131, 1044, 816, 733; ^1^H NMR (300 MHz, DMSO-*d*_6_): δ 11.73 (br s, 1H, N*H*), 8.89 (d, 1H, *J* = 1 Hz, *H*-9), 8.40 (s, 1H, *H*-2), 8.35 (dd, 1H, *J_1_* = 1 Hz, *J_2_* = 8 Hz, *H*-7), 7.85 (d, 1H, *J* = 8 Hz, *H*-6), 7.22 (s, 2H, N*H*_2_); ^13^C NMR (75 MHz, DMSO-*d*_6_): δ 151.4, 151.3, 142.6, 141.4, 140.5, 122.5, 120.4, 119.4, 117.2, 113.3; HRMS calcd for C_10_H_8_N_5_O_2_ [M + H]^+^ 230.0678 found 230.0682.

*5-Methyl-8-nitro-5H-pyrimido[5,4-b]indol-4-amine* (**2b**): yellow powder (0.182 g, 75%) obtained from **8b** after 40 min of irradiation at 170 °C according to the general procedure; mp > 320 °C; IR (neat) ν_max_(cm^−1^): 3087, 1617, 1585, 1510, 1472, 1327, 1302, 1259, 1235, 1083, 1026, 915, 844, 791, 729; ^1^H NMR (300 MHz, DMSO-*d*_6_): δ 8.89 (d, 1H, *J* = 1 Hz, *H*-9), 8.40 (dd, 1H, *J_1_* = 1 Hz, *J_2_* = 8 Hz, *H*-7), 8.39 (s, 1H, *H*-2), 7.90 (d, 1H, *J* = 8 Hz, *H*-6), 7.21 (s, 2H, N*H*_2_), 4.14 (s, 3H, C*H*_3_); ^13^C NMR (75 MHz, DMSO-*d*_6_): δ 151.9, 151.1, 143.5, 143.0, 140.3, 122.7, 120.5, 119.7, 117.1, 111.3, 32.4; HRMS calcd for C_11_H_10_N_5_O_2_ [M + H]^+^ 244.0834 found 244.0827.

*5-Ethyl-8-nitro-5H-pyrimido[5,4-b]indol-4-amine* (**2c**): yellow powder (0.185 g, 72%) obtained from **8c** after 30 min of irradiation at 170 °C according to the general procedure; mp 299–300 °C; IR (neat) ν_max_(cm^−1^): 3072, 1617, 1586, 1507, 1475, 1456, 1329, 1302, 1257, 1227, 1090, 944, 805, 753, 735; ^1^H NMR (300 MHz, DMSO-*d*_6_): δ 8.91 (d, 1H, *J* = 1 Hz, *H*-9), 8.42 (s, 1H, *H*-2), 8.40 (dd, 1H, *J_1_* = 1 Hz, *J_2_* = 8 Hz, *H*-7), 7.97 (d, 1H, *J* = 8 Hz, *H*-6), 7.23 (s, 2H, N*H*_2_), 4.70 (q, 2H, *J* = 7 Hz, C*H*_2_), 1.27 (t, 3H, *J* = 7 Hz, C*H*_3_); ^13^C NMR (75 MHz, DMSO-*d*_6_): δ 152.4, 152.3, 144.1, 142.3, 140.5, 122.9, 120.1, 119.3, 117.2, 111.3, 40.1, 16.1; HRMS calcd for C_12_H_12_N_5_O_2_ [M + H]^+^ 258.0991 found 258.0986.

*5-Benzyl-8-nitro-5H-pyrimido[5,4-b]indol-4-amine* (**2d**): yellow powder (0.252 g, 79%) obtained from **8d** after 30 min of irradiation at 170 °C according to the general procedure; mp > 320 °C; IR (neat) ν_max_(cm^−1^): 3052, 1643, 1620, 1586, 1513, 1473, 1451, 1400, 1331, 1315, 1203, 1076, 1045, 939, 804, 791, 732; ^1^H NMR (300 MHz, DMSO-*d*_6_): δ 8.94 (d, 1H, *J* = 1 Hz, *H*-9), 8.44 (s, 1H, *H*-2), 8.41 (dd, 1H, *J_1_* = 1 Hz, *J_2_* = 8 Hz, *H*-7), 8.03 (d, 1H, *J* = 8 Hz, *H*-6), 7.28-7.18 (m, 5H, N*H*_2_, and *H*-ar), 7.01 (dd, 2H, *J_1_* = 1 Hz, *J_2_* = 8 Hz, *H*-ar), 5.99 (s, 2H, C*H*_2_); ^13^C NMR (75 MHz, DMSO-*d*_6_): δ 151.5 (2C), 144.3, 143.3, 140.9, 137.4, 128.7 (2C), 127.5, 126.1 (2C), 123.2, 120.4, 119.7, 117.2, 111.9, 47.4; HRMS calcd for C_17_H_14_N_5_O_2_ [M + H]^+^ 320.1147 found 320.1145.

*9H-Pyrimido[4,5-b]indol-4-amine* (**3a**): cream-coloured powder (0.131 g, 71%) obtained from *N’*-(3-cyano-1*H*-indol-2-yl)-*N,N*-dimethylformimidamide **9a** after 40 min of irradiation at 200 °C according to the general procedure; mp > 320 °C; IR (neat) ν_max_(cm^−1^): 3068, 1637, 1624, 1604, 1580, 1569, 1302, 1255, 981, 798, 750, 705; ^1^H NMR (300 MHz, DMSO-*d*_6_): δ 11.81 (br s, 1H, N*H*), 8.27 (d, 1H, *J* = 8 Hz, *H*-5), 8.24 (s, 1H, *H*-2), 7.43 (d, 1H, *J* = 8 Hz, *H*-8), 7.34 (td, 1H, *J_1_* = 1 Hz, *J_2_* = 8 Hz, *H*-7), 7.22 (td, 1H, *J_1_* = 1 Hz, *J_2_* = 8 Hz, *H*-6), 7.13 (s, 2H, N*H*_2_); ^13^C NMR (75 MHz, DMSO-*d*_6_): δ 157.6, 155.6, 154.8, 136.2, 124.4, 121.2, 120.0, 119.8, 110.8, 95.2; HRMS calcd for C_10_H_9_N_4_ [M + H]^+^ 185.0827 found 185.0820.

*9-Methyl-9H-pyrimido[4,5-b]indol-4-amine* (**3b**): brown powder (0.168 g, 85%) obtained from **9b** after 30 min of irradiation at 200 °C according to the general procedure; mp 189–190 °C; IR (neat) ν_max_(cm^−1^): 3059, 1622, 1588, 1556, 1507, 1463, 1449, 1326, 1309, 1300, 1194, 978, 795, 731; ^1^H NMR (300 MHz, DMSO-*d*_6_): δ 8.32 (d, 1H, *J* = 8 Hz, *H*-5), 8.31 (s, 1H, *H*-2), 7.59 (d, 1H, *J* = 8 Hz, *H*-8), 7.43 (td, 1H, *J_1_* = 1 Hz, *J_2_* = 8 Hz, *H*-7), 7.29 (td, 1H, *J_1_* = 1 Hz, *J_2_* = 8 Hz, *H*-6), 7.22 (s, 2H, N*H*_2_), 3.82 (s, 3H, C*H*_3_); ^13^C NMR (75 MHz, DMSO-*d*_6_): δ 157.7, 155.2, 154.9, 137.5, 124.7, 124.4, 121.2, 120.3, 119.3, 109.2, 28.1; HRMS calcd for C_11_H_11_N_4_ [M + H]^+^ 199.0984 found 199.0974.

*9-Ethyl-9H-pyrimido[4,5-b]indol-4-amine* (**3c**): brown powder (0.204 g, 96%) obtained from **9c** after 30 min of irradiation at 200 °C according to the general procedure; mp 137–138 °C; IR (neat) ν_max_(cm^−1^): 3062, 1623, 1558, 1448, 1464, 1329, 1113, 798, 736, 708; ^1^H NMR (300 MHz, DMSO-*d*_6_): δ 8.34 (d, 1H, *J* = 8 Hz, *H*-5), 8.32 (s, 1H, *H*-2), 7.63 (d, 1H, *J* = 8 Hz, *H*-8), 7.45 (td, 1H, *J_1_* = 1 Hz, *J_2_* = 8 Hz, *H*-7), 7.26 (td, 1H, *J_1_* = 1 Hz, *J_2_* = 8 Hz, *H*-6), 7.21 (s, 2H, N*H*_2_), 4.41 (q, 2H, *J* = 7 Hz, C*H*_2_), 1.31 (t, 3H, *J* = 7 Hz, C*H*_3_); ^13^C NMR (75 MHz, DMSO-*d*_6_): δ 157.5, 154.8, 154.5, 136.3, 124.5, 121.4, 120.3, 119.6, 109.3, 94.8, 35.7, 14.0; HRMS calcd for C_12_H_13_N_4_ [M + H]^+^ 213.1140 found 213.1130.

*9-Benzyl-9H-pyrimido[4,5-b]indol-4-amine* (**3d**): cream-coloured powder (0.184 g, 67%) obtained from **9d** after 30 min of irradiation at 200 °C according to the general procedure; mp 217–218 °C; IR (neat) ν_max_(cm^−1^): 3051, 1620, 1585, 1568, 1556, 1458, 1445, 1428, 1297, 799, 753, 740; ^1^H NMR (300 MHz, DMSO-*d*_6_): δ 8.34 (d, 1H, *J* = 8 Hz, *H*-5), 8.33 (s, 1H, *H*-2), 7.55 (d, 1H, *J* = 8 Hz, *H*-8), 7.37 (td, 1H, *J_1_* = 1 Hz, *J_2_* = 8 Hz, *H*-7), 7.29-7.21 (m, 8H, *H*-6, N*H*_2_, and *H*-ar), 5.60 (s, 2H, C*H*_2_); ^13^C NMR (75 MHz, DMSO-*d*_6_): δ 157.6, 155.0, 137.5, 136.6, 128.5 (2C), 127.3, 127.1 (2C), 124.6, 121.4, 120.6, 119.6, 117.2, 109.8, 94.8, 44.0; HRMS calcd for C_17_H_15_N_4_ [M + H]^+^ 275.1297 found 275.1292.

*7-Nitro-9H-pyrimido[4,5-b]indol-4-amine* (**4a**): brown powder (0.153 g, 67%) obtained from **10a** after 30 min of irradiation at 200 °C according to the general procedure; mp > 320 °C; IR (neat) ν_max_(cm^−1^): 3052, 1621, 1529, 1500, 1471, 1375, 1304, 1287, 1257, 1204, 1124, 1071, 884, 820, 753, 734; ^1^H NMR (300 MHz, DMSO-*d*_6_): δ 12.42 (br s, 1H, N*H*), 8.53 (d, 1H, *J* = 9 Hz, *H*-5), 8.34 (s, 1H, *H*-2), 8.25 (d, 1H, *J* = 2 Hz, *H*-8), 8.11 (dd, 1H, *J_1_* = 2 Hz, *J_2_* = 9 Hz, *H*-6), 7.62 (s, 2H, N*H*_2_); ^13^C NMR (75 MHz, DMSO-*d*_6_): δ 158.4, 157.9, 156.8, 143.8, 135.4, 125.5, 121.2, 115.2, 106.5, 94.9; HRMS calcd for C_10_H_8_N_5_O_2_ [M + H]^+^ 230.0678 found 230.0689.

*9-Methyl-7-nitro-9H-pyrimido[4,5-b]indol-4-amine* (**4b**): yellow powder (0.195 g, 80%) obtained from **10b** after 30 min of irradiation at 200 °C according to the general procedure *G*; mp 309–310 °C; IR (neat) ν_max_(cm^−1^): 3031, 1568, 1513, 1482, 1331, 1309, 1284, 1273, 1193, 987, 875, 853, 811, 726; ^1^H NMR (300 MHz, DMSO-*d*_6_): δ 8.55 (d, 1H, *J* = 9 Hz, *H*-5), 8.51 (d, 1H, *J* = 2 Hz, *H*-8), 8.39 (s, 1H, *H*-2), 8.12 (dd, 1H, *J_1_* = 2 Hz, *J_2_* = 9 Hz, *H*-6), 7.67 (s, 2H, N*H*_2_), 3.91 (s, 3H, C*H*_3_); ^13^C NMR (75 MHz, DMSO-*d*_6_): δ 158.2, 157.3, 156.7, 144.0, 136.7, 124.9, 121.1, 115.5, 105.5, 94.5, 27.9; HRMS calcd for C_11_H_10_N_5_O_2_ [M + H]^+^ 244.0834 found 244.0831.

*9-Ethyl-7-nitro-9H-pyrimido[4,5-b]indol-4-amine* (**4c**): brown powder (0.162 g, 63%) obtained from **10c** after 30 min of irradiation at 200 °C according to the general procedure; mp 285–286 °C; IR (neat) ν_max_(cm^−1^): 3109, 1618, 1582, 1563, 1509, 1485, 1448, 1322, 1276, 1184, 1135, 820, 743, 732; ^1^H NMR (300 MHz, DMSO-*d*_6_): δ 8.58 (d, 1H, *J* = 2 Hz, *H*-8), 8.55 (d, 1H, *J* = 9 Hz, *H*-5), 8.39 (s, 1H, *H*-2), 8.12 (dd, 1H, *J_1_* = 2 Hz, *J_2_* = 9 Hz, *H*-6), 7.69 (s, 2H, N*H*_2_), 4.53 (q, 2H, *J* = 7 Hz, C*H*_2_), 1.32 (t, 3H, *J* = 7 Hz, C*H*_3_); ^13^C NMR (75 MHz, DMSO-*d*_6_): δ 158.3, 156.8, 156.7, 144.1, 135.6, 125.1, 121.3, 115.4, 105.4, 94.5, 36.2, 14.1; HRMS calcd for C_12_H_12_N_5_O_2_ [M + H]^+^ 258.0991 found 258.0999.

*9-Benzyl-7-nitro-9H-pyrimido[4,5-b]indol-4-amine* (**4d**): yellow powder (0.214 g, 67%) obtained from **10d** after 30 min of irradiation at 200 °C according to the general procedure; mp 241–242 °C; IR (neat) ν_max_(cm^−1^): 3031, 1558, 1508, 1477, 1448, 1319, 1268, 1170, 1086, 801, 733; ^1^H NMR (300 MHz, DMSO-*d*_6_): δ 8.60 (d, 1H, *J* = 2 Hz, *H*-8), 8.49 (d, 1H, *J* = 9 Hz, *H*-5), 8.43 (s, 1H, *H*-2), 8.13 (dd, 1H, *J_1_* = 2 Hz, *J_2_* = 9 Hz, *H*-6), 7.76 (s, 2H, N*H*_2_), 7.32-7.21 (m, 5H, *H*-ar), 4.53 (s, 2H, C*H*_2_); ^13^C NMR (75 MHz, DMSO-*d*_6_): δ 158.4, 157.4, 156.9, 144.1, 137.0, 135.9, 128.7 (2C), 127.5, 127.0 (2C), 125.3, 121.4, 115.8, 105.7, 94.5, 44.2; HRMS calcd for C_17_H_14_N_5_O_2_ [M + H]^+^ 320.1147 found 320.1133.

#### 4.2.2. General Procedure for the Synthesis of *N*-alkylated Harmine Derivatives (Compounds **11b**–**d**).

A mixture of starting harmine (**11a**, 212 mg, 1 mmol) and corresponding DMF-dialkylacetal (DMF-DMA, DMF-DEA, or DMF-DBA) (10 mmol) in DMF (10 mmol) was irradiated (atmospheric pressure) at various temperatures (800 W). On completion, the solution was cooled to room temperature and crude products were extracted with ethylacetate. The organic layers were washed with cold water, dried over Na_2_SO_4_, filtered, and evaporated in vacuo. Purification by silica gel column chromatography using a gradient of dichloromethane/ethylacetate (100:0 to 0:100, *v*/*v*) as the eluent gave the desired products.

*7-Methoxy-1,9-dimethyl-β-carboline* (**11b**): Yield: 79%, obtained from DMF-DMA after 60 min of irradiation at 140 °C according to the general procedure; mp 125–126 °C; ^1^H NMR (300 MHz, DMSO-*d*_6_): δ 8.14 (d, 1H, *J* = 5 Hz, *H*-3), 8.09 (d, 1H, *J* = 8 Hz, *H*-5), 7.86 (d, 1H, *J* = 5 Hz, *H*-4), 7.20 (d, 1H, *J* = 2 Hz, *H*-8), 6.86 (dd, 1H, *J_1_* = 2 Hz, *J_2_* = 8 Hz, *H*-6), 4.13 (s, 3H, NC*H*_3_), 3.92 (s, 3H, OC*H*_3_), 3.01 (s, 3H, C*H*_3_); HRMS calcd for C_14_H_15_N_2_O [M + H]^+^ 227.1184 found 227.1180.

*9-Ethyl-7-methoxy-1-methyl-β-carboline* (**11c**): Yield: 80%, obtained from DMF-DEA after 60 min of irradiation at 160 °C according to the general procedure; mp 109–110 °C; ^1^H NMR (300 MHz, DMSO-*d*_6_): δ 8.16 (d, 1H, *J* = 5 Hz, *H*-3), 8.09 (d, 1H, *J* = 8 Hz, *H*-5), 7.87 (d, 1H, *J* = 5 Hz, *H*-4), 7.20 (d, 1H, *J* = 2 Hz, *H*-8), 6.87 (dd, 1H, *J_1_* = 2 Hz, *J_2_* = 8 Hz, *H*-6), 4.62 (q, 2H, *J* = 7 Hz, C*H*_2_), 3.92 (s, 3H, OC*H*_3_), 2.96 (s, 3H, C*H*_3_), 1.35 (t, 3H, *J* = 7 Hz, CH_2_C*H*_3_); HRMS calcd for C_15_H_17_N_2_O [M + H]^+^ 241.1341 found 241.1338.

*9-Benzyl-7-methoxy-1-methyl-β-carboline* (**11d**): Yield: 62%, obtained from DMF-DBA after 60 min of irradiation at 160 °C according to the general procedure; mp 131–132 °C; ^1^H NMR (300 MHz, DMSO-*d*_6_): δ 8.18 (d, 1H, *J* = 5 Hz, *H*-3), 8.14 (d, 1H, *J* = 8 Hz, *H*-5), 7.94 (d, 1H, *J* = 5 Hz, *H*-4), 7.38-7.19 (m, 5H, *H*-8, and *H*-ar), 6.94-6.89 (m, 2H, *H*-6, and *H*-ar), 5.90 (s, 2H, C*H*_2_), 3.82 (s, 3H, OC*H*_3_), 2.74 (s, 3H, C*H*_3_); HRMS calcd for C_20_H_19_N_2_O [M + H]^+^ 303.1497 found 303.1499.

### 4.3. In Vitro Kinase Preparation and Assays

Buffers, kinase preparations, and assays were performed as described in ref. [12,13,14] according methods initially published in previous works [34,35,36].

## Figures and Tables

**Table 1:**
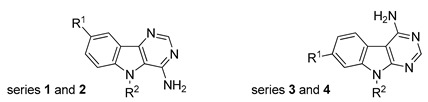
Synthesis of series **1**, **2**, **3**, and **4**: temperature, reaction time, and isolated yields.

Starting Indole	Temperature (°C)	Time (min)	Product	R^1^	R^2^	Yield (%)
**7a**	170	30	**1a**	H	H	81
**7b**	170	30	**1b**	H	Me	58
**7c**	170	30	**1c**	H	Et	64
**7d**	170	40	**1d**	H	Bn	52
**8a**	170	60	**2a**	NO_2_	H	65
**8b**	170	40	**2b**	NO_2_	Me	75
**8c**	170	30	**2c**	NO_2_	Et	72
**8d**	170	30	**2d**	NO_2_	Bn	79
**9a**	200	40	**3a**	H	H	71
**9b**	200	30	**3b**	H	Me	85
**9c**	200	30	**3c**	H	Et	96
**9d**	200	30	**3d**	H	Bn	67
**10a**	200	30	**4a**	NO_2_	H	67
**10b**	200	30	**4b**	NO_2_	Me	80
**10c**	200	30	**4c**	NO_2_	Et	63
**10d**	200	30	**4d**	NO_2_	Bn	67

**Table 2:** Synthesis of compounds **11b**–**d**: experimental conditions and isolated yields.

*N,N*-dimethylformamide (DMF)-dialkylacetal (R)	Temperature (°C)	Product	Yield (%)
DMF-DMA (Me)	140	**11b**	79
DMF-DEA (Et)	160	**11c**	80
DMF-DBA (Bn)	160	**11d**	62

**Table 3:**
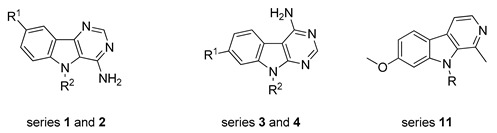
Kinase inhibitory potencies (IC_50_ in μM) ^1^ for compounds of the series 1, 2, 3, and 4.

Compound	R^1^	R^2^	CDK5/p25	CK1δ/ε	DYRK1A	GSK-3α/β
**1a**	H	H	>10	2.0	2.2	>10
**1b**	H	Me	>10	4.0	5.8	>10
**1c**	H	Et	>10	2.8	4.1	>10
**1d**	H	Bn	>10	**0.6**	>10	>10
**2a**	NO_2_	H	>10	>10	7.6	>10
**2b**	NO_2_	Me	>10	>10	>10	>10
**2c**	NO_2_	Et	>10	>10	>10	>10
**2d**	NO_2_	Bn	>10	>10	>10	>10
**3a**	H	H	6	**0.7**	3.1	>10
**3b**	H	Me	>10	2.5	>10	>10
**3c**	H	Et	>10	1.6	9.8	>10
**3d**	H	Bn	>10	2.7	>10	>10
**4a**	NO_2_	H	>10	3.5	7.6	>10
**4b**	NO_2_	Me	>10	2.8	>10	>10
**4c**	NO_2_	Et	>10	1.6	5.9	>10
**4d**	NO_2_	Bn	>10	>10	>10	>10
**11a** (Harmine)	R = H		>10	1.5	**0.029**	>10
**11b**	R = Me		>10	>10	**0.13**	>10
**11c**	R = Et		>10	>10	**0.037**	>10
**11d**	R = Bn		>10	>10	**0.059**	>10

^1^ Average of triplicate determination (<10% variation among values).

## Supplementary Materials

The following materials are available online at https://www.mdpi.com/1424-8247/13/5/89/s1. Figures S1–S16. 1H- & 13C-NMR Spectra for Compounds **1a**–**d**, **2a**–**d**, **3a**–**d** and **4a**–**d**.

## References

[1] Manning G., Whyte D.B., Martinez R., Hunter T., Sudarsanam S. (2002). The Protein Kinase Complement of the Human Genome. Science.

[2] Ionescu A., Dufrasne F., Gelbcke M., Jabin I., Kiss R., Lamoral-Theys D. (2012). DYRK1A kinase inhibitors with emphasis on cancer. Mini Rev. Med. Chem..

[3] Fernández-Martínez P., Zahonero C., Sánchez-Gómez P. (2015). DYRK1A: The double-edged kinase as a protagonist in cell growth and tumorigenesis. Mol. Cell. Oncol..

[4] Duchon A., Herault Y. (2016). DYRK1A, a Dosage-Sensitive Gene Involved in Neurodevelopmental Disorders, Is a Target for Drug Development in Down Syndrome. Front. Behav. Neurosci..

[5] Branca C., Shaw D.M., Belfiore R., Gokhale V., Shaw A.Y., Foley C., Smith B., Hulme C., Dunckley T., Meechoovet B. (2017). Dyrk1 inhibition improves Alzheimer’s disease-like pathology. Aging Cell.

[6] Stotani S., Giordanetto F., Medda F. (2016). DYRK1A inhibition as potential treatment for Alzheimer’s disease. Future Med. Chem..

[7] Roskoski R. (2020). Properties of FDA-approved small molecule protein kinase inhibitors: A 2020 update. Pharmacol. Res..

[8] Alexandre F.-R., Berecibar A., Wrigglesworth R., Besson T. (2003). Efficient synthesis of thiazoloquinazolinone derivatives. Tetrahedron Lett..

[9] Loidreau Y., Besson T. (2011). Microwave-assisted thermal decomposition of formamide: A tool for coupling a pyrimidine ring with an aromatic partner. Tetrahedron.

[10] Foucourt A., Dubouilh-Benard C., Chosson E., Corbiere C., Buquet C., Iannelli M., Leblond B., Marsais F., Besson T. (2010). Microwave-accelerated Dimroth rearrangement for the synthesis of 4-anilino-6-nitroquinazolines. Application to an efficient synthesis of a microtubule destabilizing agent. Tetrahedron.

[11] Loidreau Y., Dubouilh-Benard C., Marchand P., Nourrisson M.-R., Duflos M., Buquet C., Corbière C., Besson T. (2013). Efficient New Synthesis ofN-Arylbenzo [b] furo [3,2-d] pyrimidin-4-amines and Their Benzo [b] thieno [3,2-d] pyrimidin-4-amine Analogues via a Microwave-Assisted Dimroth Rearrangement. J. Heterocycl. Chem..

[12] Loidreau Y., Marchand P., Dubouilh-Benard C., Nourrisson M.-R., Duflos M., Loaëc N., Meijer L., Besson T. (2013). Synthesis and biological evaluation of N-aryl-7-methoxybenzo [b] furo [3,2-d] pyrimidin-4-amines and their N-arylbenzo [b] thieno [3,2-d] pyrimidin-4-amine analogues as dual inhibitors of CLK1 and DYRK1A kinases. Eur. J. Med. Chem..

[13] Loidreau Y., Marchand P., Dubouilh-Benard C., Nourrisson M.-R., Duflos M., Lozach O., Loaëc N., Meijer L., Besson T. (2012). Synthesis and biological evaluation of N-arylbenzo[b]thieno[3,2-d]pyrimidin-4-amines and their pyrido and pyrazino analogues as Ser/Thr kinase inhibitors. Eur. J. Med. Chem..

[14] Loidreau Y., Deau E., Marchand P., Nourrisson M.-R., Logé C., Coadou G., Loaëc N., Meijer L., Besson T. (2015). Synthesis and molecular modelling studies of 8-arylpyrido[3′,2′:4,5]thieno[3,2-d]pyrimidin-4-amines as multitarget Ser/Thr kinases inhibitors. Eur. J. Med. Chem..

[15] Jarhad D.B., Mashelkar K.K., Kim H.-R., Noh M., Jeong L.S. (2018). Dual-Specificity Tyrosine Phosphorylation-Regulated Kinase 1A (DYRK1A) Inhibitors as Potential Therapeutics. J. Med. Chem..

[16] Czarna A., Wang J., Zelencova D., Liu Y., Deng X., Choi H.G., Zhang T., Zhou W., Chang J.W., Kildalsen H. (2018). Novel Scaffolds for Dual Specificity Tyrosine-Phosphorylation-Regulated Kinase (DYRK1A) Inhibitors. J. Med. Chem..

[17] Meijer L., Flajolet M., Greengard P. (2004). Pharmacological inhibitors of glycogen synthase kinase 3. Trends Pharmacol. Sci..

[18] Ryoo S.-R., Jeong H.K., Radnaabazar C., Yoo J.-J., Cho H.-J., Lee H.-W., Kim I.-S., Cheon Y.-H., Ahn Y.S., Chung S.-H. (2007). DYRK1A-mediated Hyperphosphorylation of Tau. J. Boil. Chem..

[19] Vasquez A.A., Aulchenko Y.S., Isaacs A., Van Oosterhout A., Sleegers K., Hofman A., Van Broeckhoven C., Oostra B.A., Breteler M., Van Duijn C.M. (2008). Cyclin-dependent kinase 5 is associated with risk for Alzheimer’s disease in a Dutch population-based study. J. Neurol..

[20] Cozza G., Pinna L.A. (2015). Casein kinases as potential therapeutic targets. Expert Opin. Ther. Targets.

[21] Li G., Yin H., Kuret J. (2004). Casein Kinase 1 Delta Phosphorylates Tau and Disrupts Its Binding to Microtubules. J. Boil. Chem..

[22] Smith B., Medda F., Gokhale V., Dunckley T., Hulme C. (2012). Recent Advances in the Design, Synthesis, and Biological Evaluation of Selective DYRK1A Inhibitors: A New Avenue for a Disease Modifying Treatment of Alzheimer’s?. ACS Chem. Neurosci..

[23] Becker W., Joost H.-G. (1999). Structural and functional characteristics of Dyrk, a novel subfamily of protein kinases with dual specificity. Prog. Nucleic Acid Res. Mol. Biol..

[24] Aranda S., Laguna A., De La Luna S. (2010). DYRK family of protein kinases: Evolutionary relationships, biochemical properties, and functional roles. FASEB J..

[25] Tahtouh T., Elkins J.M., Filippakopoulos P., Soundararajan M., Burgy G., Durieu E., Cochet C., Schmid R., Lo N.C., Delhommel F. (2012). Selectivity, Cocrystal Structures, and Neuroprotective Properties of Leucettines, a Family of Protein Kinase Inhibitors Derived from the Marine Sponge Alkaloid Leucettamine, B. J. Med. Chem..

[26] Loidreau Y., Melissen S., Levacher V., Logé C., Graton J., Le Questel J.-Y., Besson T. (2012). Study of N1-alkylation of indoles from the reaction of 2 (or 3) -aminoindole-3- (or 2) carbonitriles with DMF-dialkylacetals. Org. Biomol. Chem..

[27] Cao R., Fan W., Guo L., Ma Q., Zhang G., Li J., Chen X., Ren Z., Qiu L. (2013). Synthesis and structure–activity relationships of harmine derivatives as potential antitumor agents. Eur. J. Med. Chem..

[28] Cao R., Chen Q., Hou X., Chen H., Guan H., Ma Y., Peng W., Xu A. (2004). Synthesis, acute toxicities, and antitumor effects of novel 9-substituted β-carboline derivatives. Bioorg. Med. Chem..

[29] Balint B., Weber C., Cruzalegui F., Burbridge M., Kotschy A. (2017). Structure-based design and synthesis of Harmine derivatives with different selectivity profiles in kinase vs monoamine oxidase inhibition. Chem. Med. Chem..

[30] Du H., Tian S., Chen J., Gu H., Li N., Wang J. (2016). Synthesis and biological evaluation of N9-substituted harmine derivatives as potential anticancer agents. Bioorg. Med. Chem. Lett..

[31] Patel K., Gadewar M., Tripathi R., Prasad S., Patel D.K. (2012). A review on medicinal importance, pharmacological activity and bioanalytical aspects of beta-carboline alkaloid “Harmine”. Asian Pac. J. Trop. Biomed..

[32] Adayev T., Wegiel J., Hwang Y.-W. (2010). Harmine is an ATP-competitive inhibitor for dual-specificity tyrosine phosphorylation-regulated kinase 1A (Dyrk1A). Arch. Biochem. Biophys..

[33] Baell J.B., Street I.P., Sleebs B.E. (2012). Pyrido [3’,2’:4,5] Thieno [3,2-d] Pyrimidin-4-Ylamine Derivatives and Their Therapeutical Use. Patent.

[34] Primot A., Baratte B., Gompel M., Borgne A., Liabeuf S., Romette J.-L., Jho E.-H., Costantini F., Meijer L. (2000). Purification of GSK-3 by Affinity Chromatography on Immobilized Axin. Protein Expr. Purif..

[35] Bach S., Knockaert M., Reinhardt J., Lozach O., Schmitt S., Baratte B., Koken M., Coburn S.P., Tang L., Jiang T. (2005). Roscovitine Targets, Protein Kinases and Pyridoxal Kinase. J. Boil. Chem..

[36] Reinhardt J., Ferandin Y., Meijer L. (2007). Purification of CK1 by affinity chromatography on immobilised axin. Protein Expr. Purif..

