# Supplementary material for: Exploring Kinase Inhibition Properties of 9H-pyrimido[5,4-b]- and [4,5-b]indol-4-amine Derivatives
Source: Pharmaceuticals (Basel). 2020 May 9;13(5):89. doi: 10.3390/ph13050089 (PMC7281298; doi:10.3390/ph13050089)
Supplement: Supplementary file 1 [file pharmaceuticals-13-00089-s001.pdf]

pharmaceuticals

Supplementary Materials

Communication

Exploring kinase inhibition properties of 9*H*-pyrimido[5,4-*b*]- and [4,5-*b*]indol-4-amine derivatives

Yvonnick Loidreau [1], Carole Dubouilh-Benard [1], Marie-Renée Nourrisson [2], Nadège Loaëc [3], Laurent Meijer [3,4], Thierry Besson [1,*] and Pascal Marchand [2,*]

[1] Normandie Univ, UNIROUEN, INSA Rouen, CNRS, COBRA UMR 6014, F-76000 Rouen, France; yvonnick.loidreau@gmail.com (Y.L.); carole.dubouilh@univ-rouen.fr (C.D.B.)
[2] Université de Nantes, Cibles et médicaments des infections et du cancer, IICiMed, EA 1155, F-44000 Nantes, France; marie-renee.nourrisson@univ-nantes.fr (M.R.N.)
[3] Station Biologique de Roscoff, Protein Phosphorylation & Human Disease group, 29680 Roscoff, France ; nadege.loaec@univ-brest.fr (N.L.)
[4] Perha Pharmaceuticals, Perharidy Peninsula, 29680 Roscoff, France; meijer@perha-pharma.com (L.M.)
* Correspondence: thierry.besson@univ-rouen.fr; +33 235-522-904 (T.B.); pascal.marchan@univ-nantes.fr (P.M.); Tel.: +33 253 009 155

1. 1H- & 13C-NMR Spectra for Compounds 1a-1d, 2a-2d, 3a-3d and 4a-4d

Figure S1. 1H NMR at 300 MHz and 13C NMR at 75.4 MHz spectra, DMSO-d6, for compound **1a.**

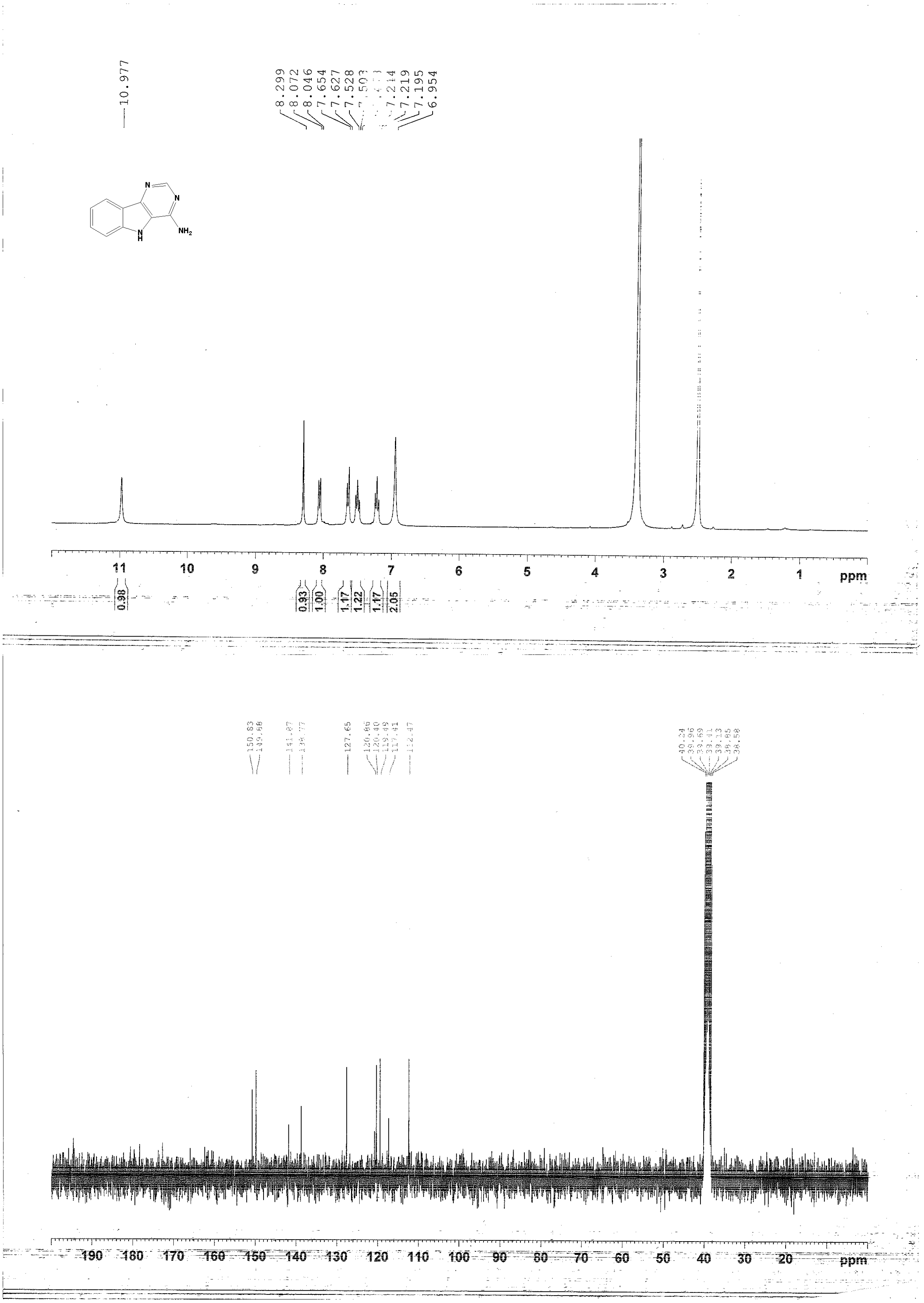

Figure S2. 1H NMR at 300 MHz and 13C NMR at 75.4 MHz spectra, DMSO-d6, for compound **1b.**

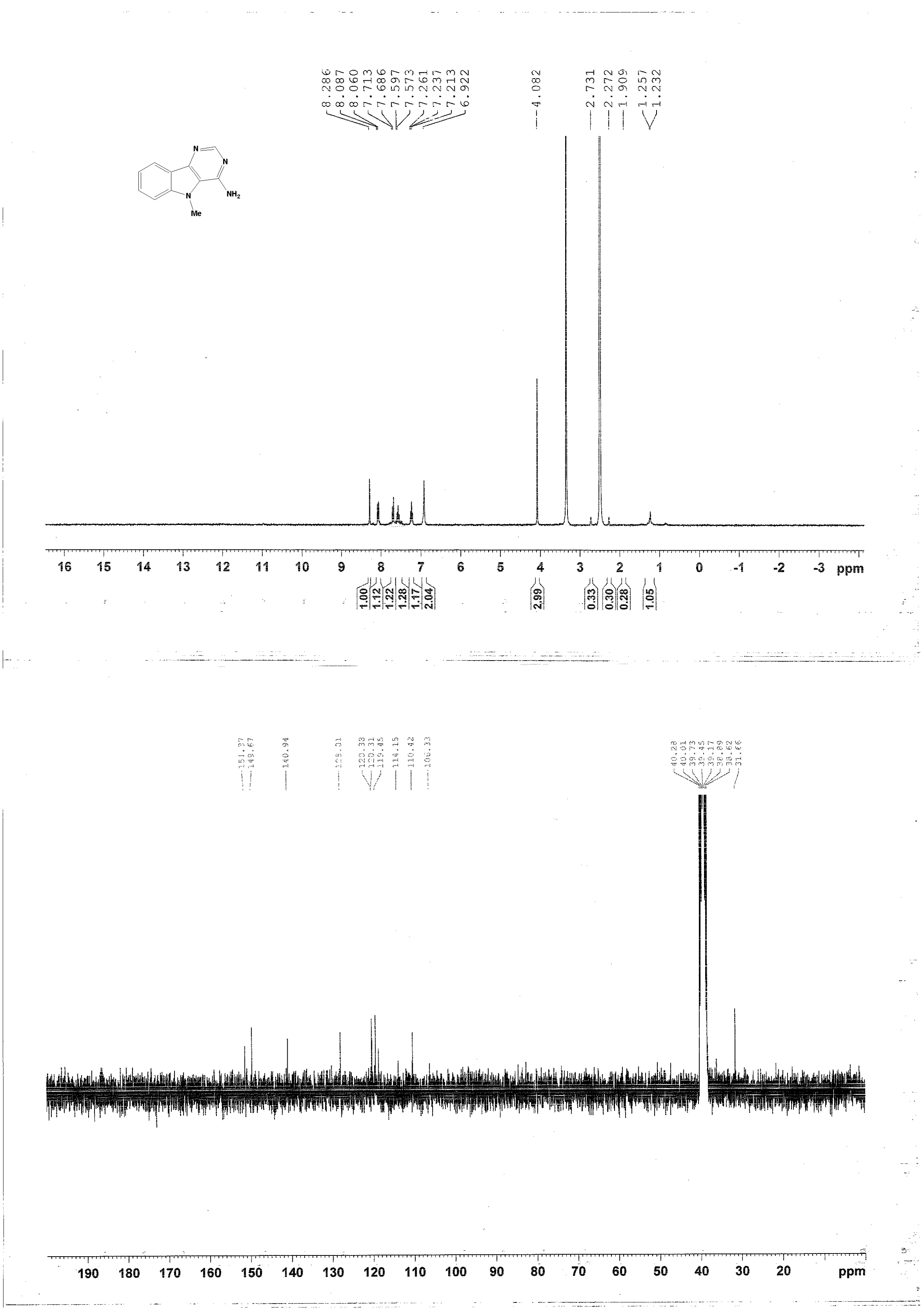

Figure S3. 1H NMR at 300 MHz and 13C NMR at 75.4 MHz spectra, DMSO-d6, for compound **1c.**

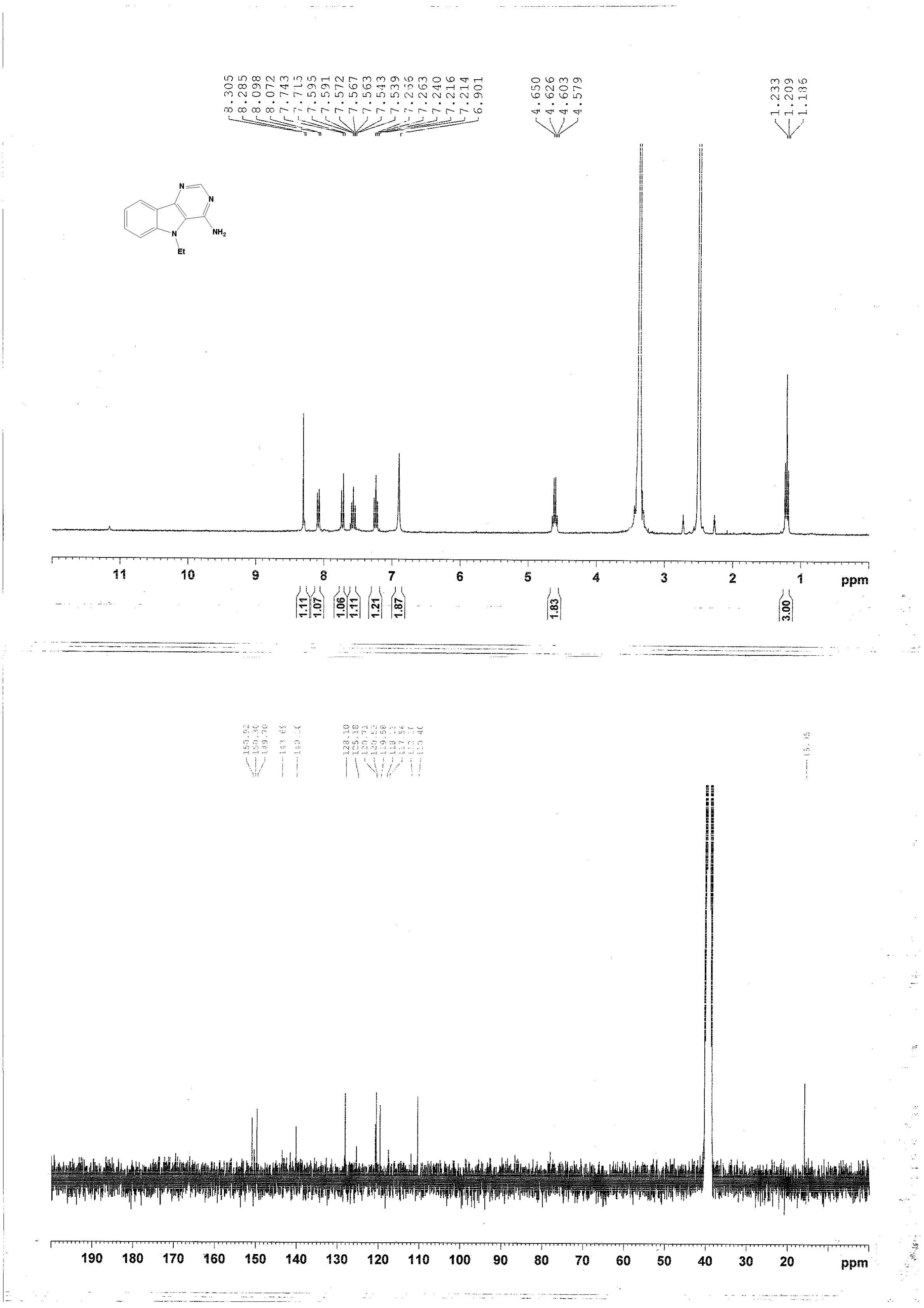

Figure S4. 1H NMR at 300 MHz and 13C NMR at 75.4 MHz spectra, DMSO-d6, for compound **1d.**

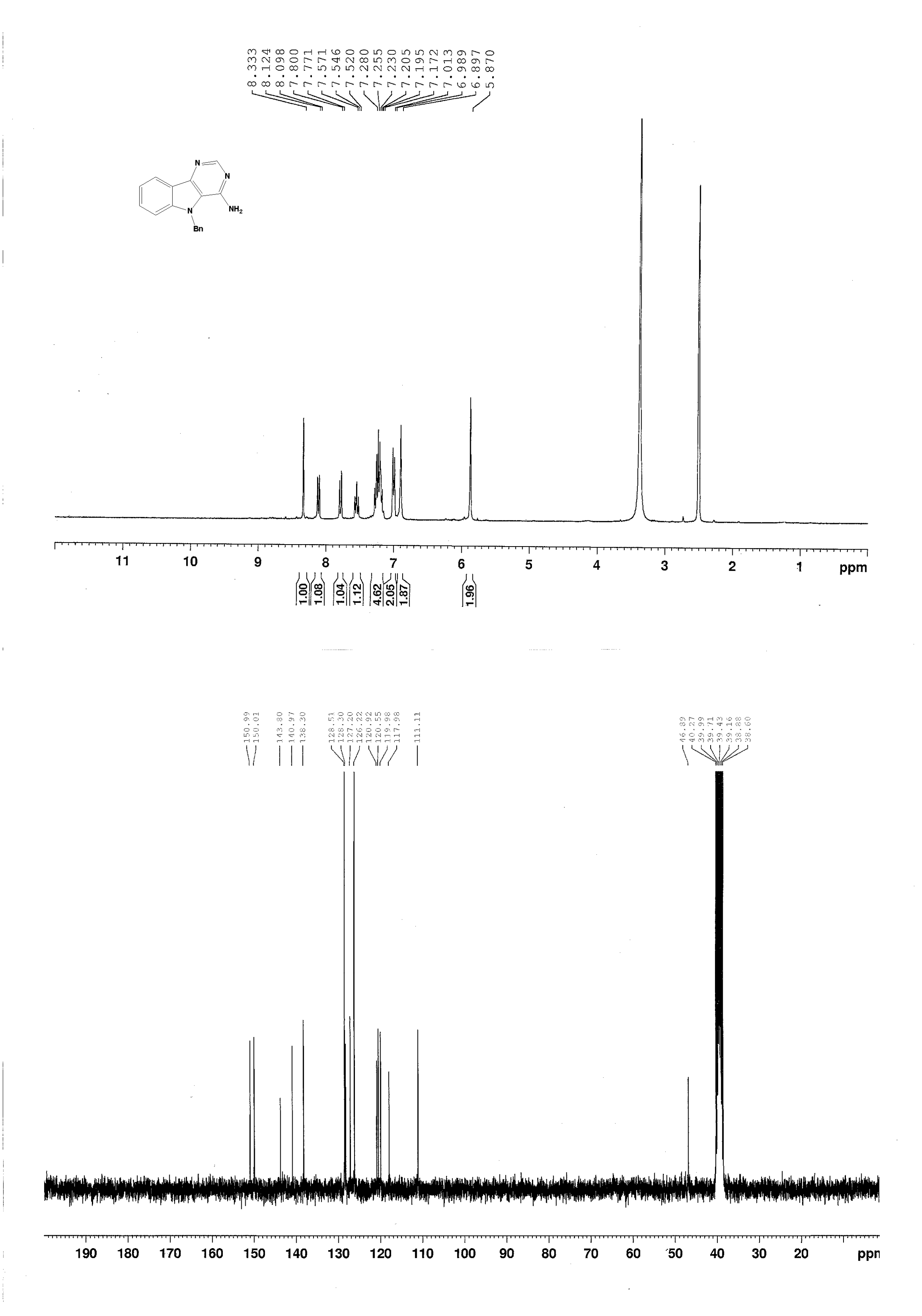

Figure S5. 1H NMR at 300 MHz and 13C NMR at 75.4 MHz spectra, DMSO-d6, for compound **2a.**

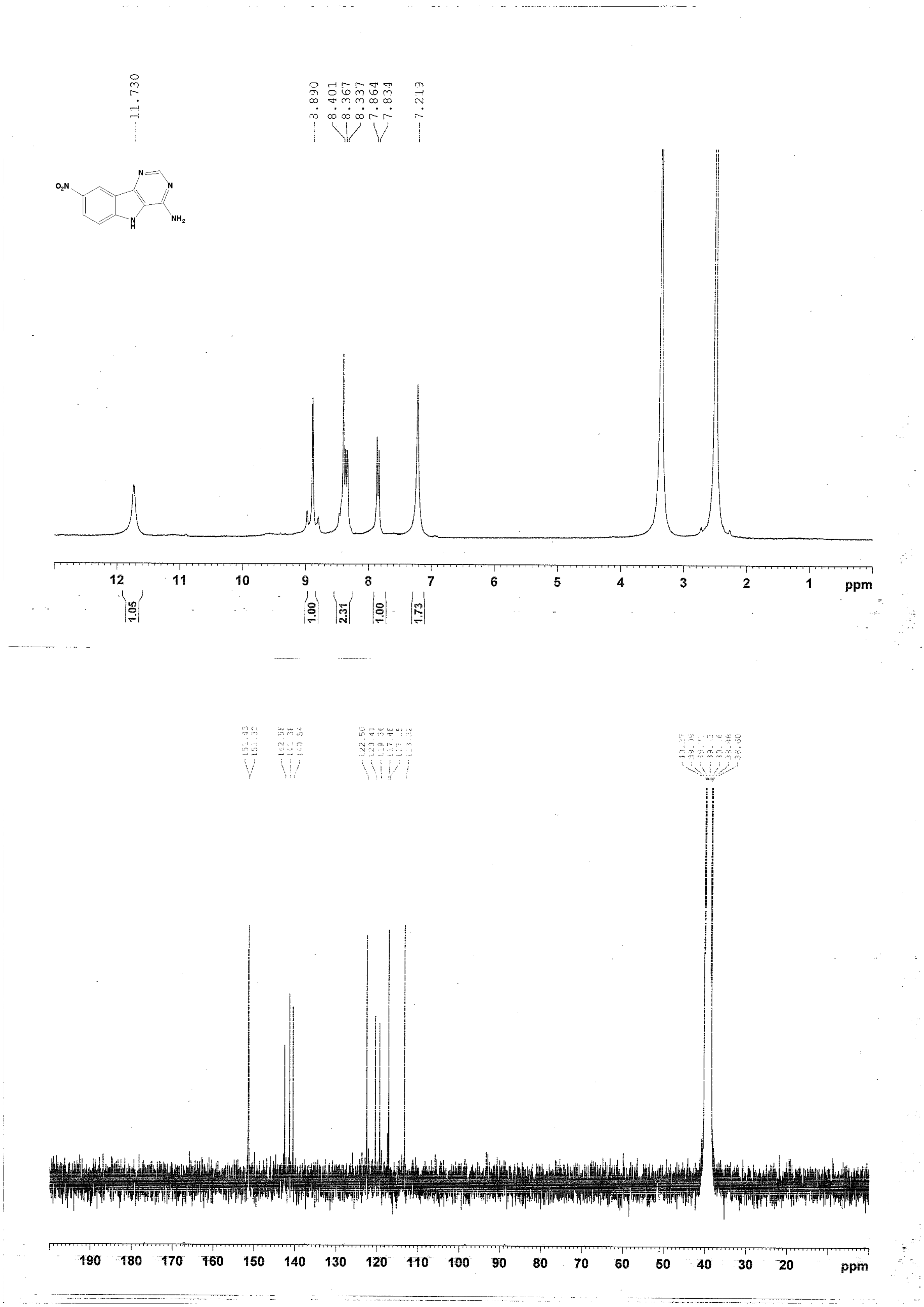

Figure S6. 1H NMR at 300 MHz and 13C NMR at 75.4 MHz spectra, DMSO-d6, for compound **2b.**

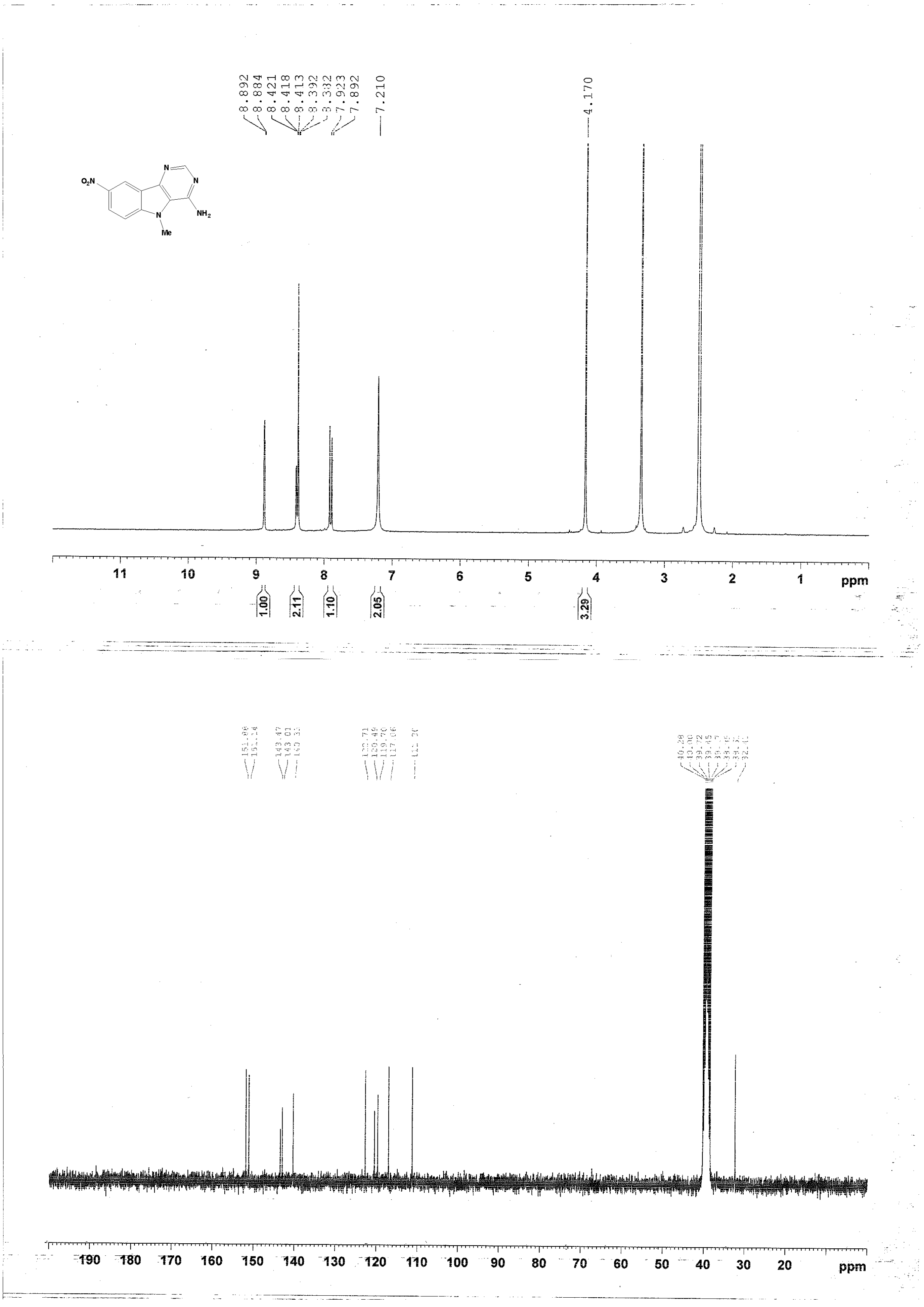

Figure S7. 1H NMR at 300 MHz and 13C NMR at 75.4 MHz spectra, DMSO-d6, for compound **2c.**

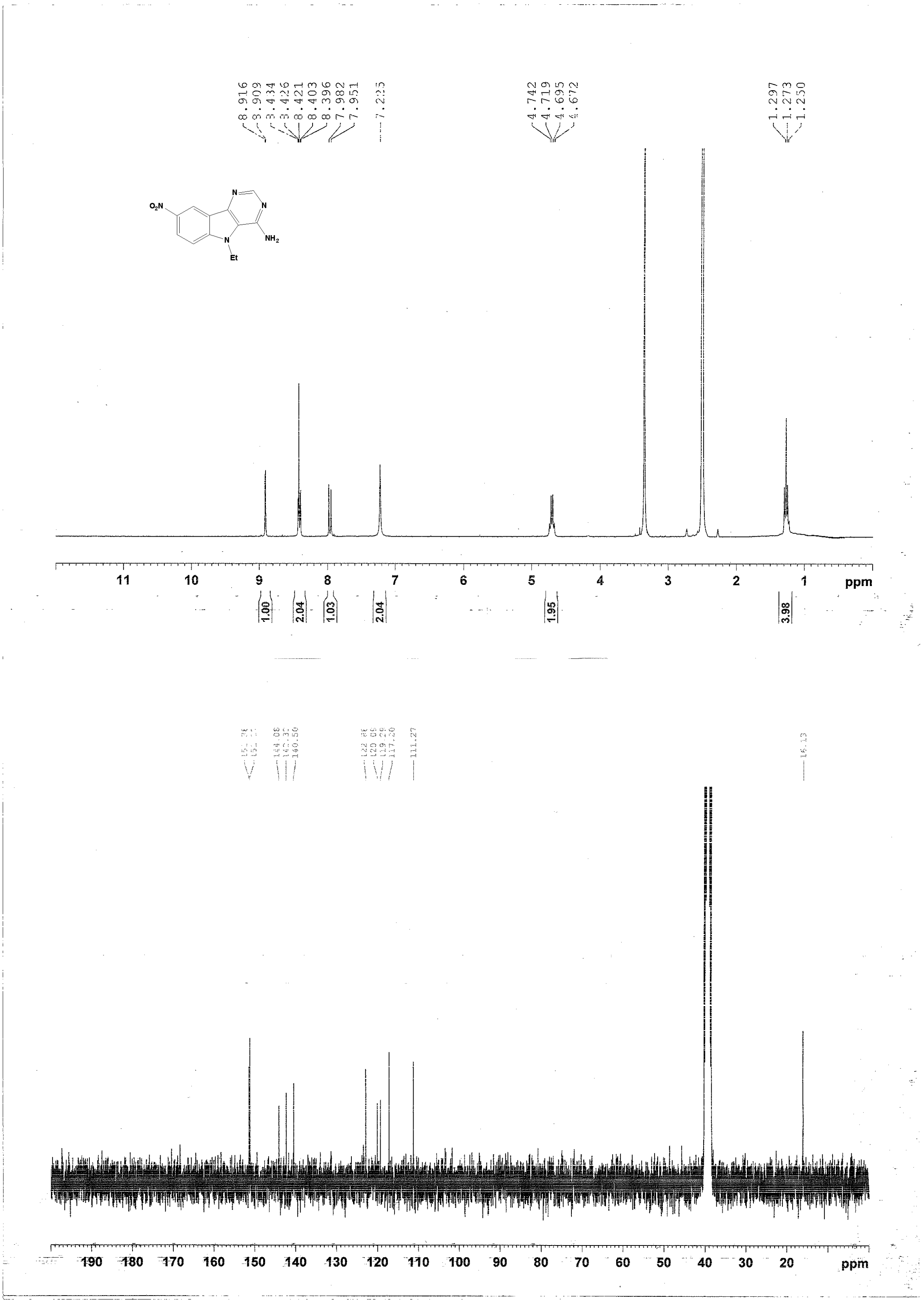

Figure S8. 1H NMR at 300 MHz and 13C NMR at 75.4 MHz spectra, DMSO-d6, for compound **2d.**

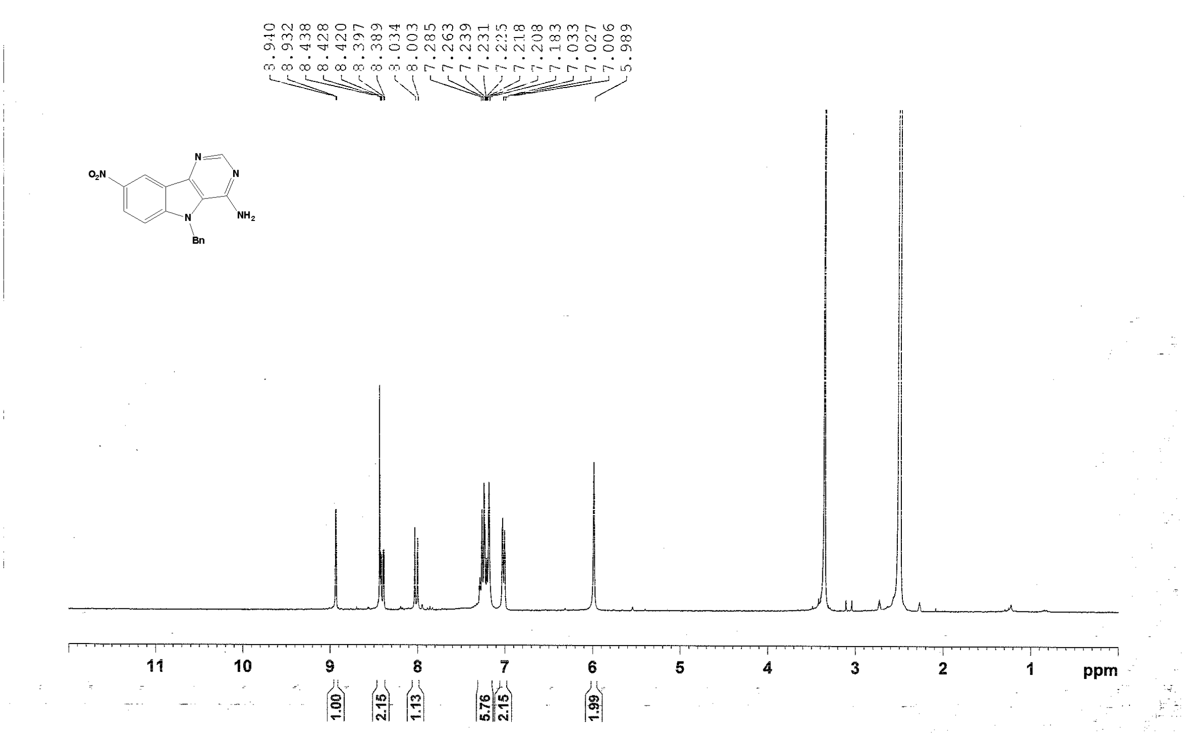

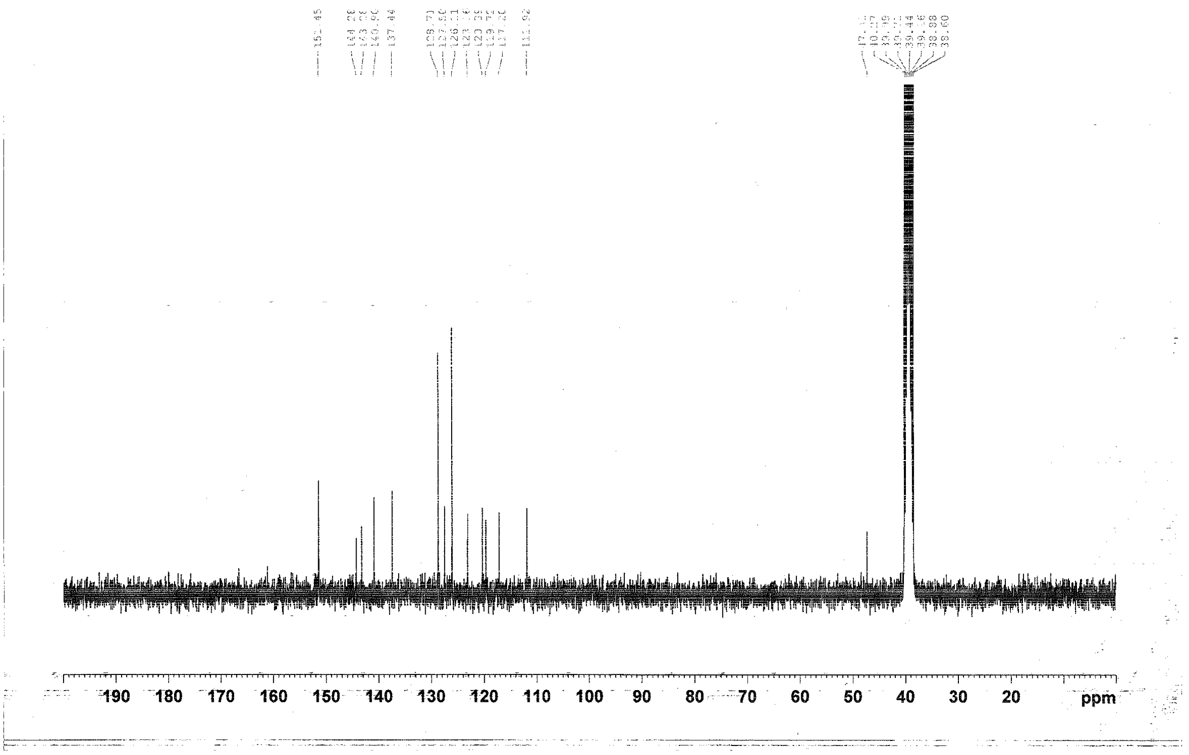

Figure S9. 1H NMR at 300 MHz and 13C NMR at 75.4 MHz spectra, DMSO-d6, for compound **3a.**

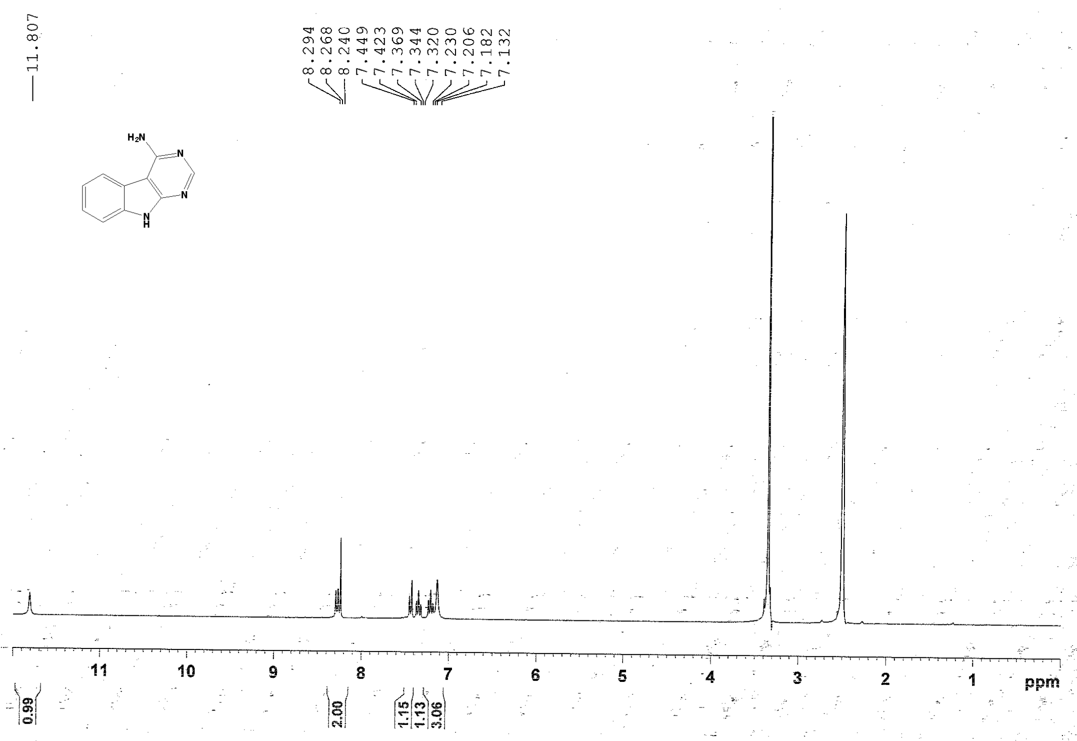

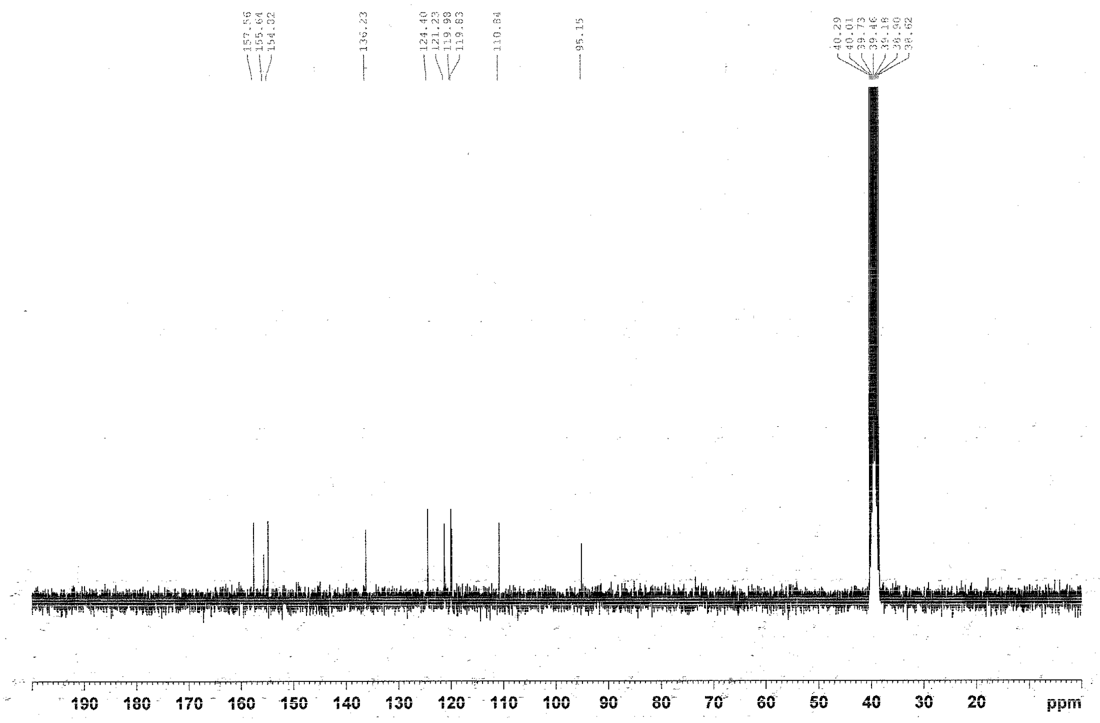

Figure S10. 1H NMR at 300 MHz and 13C NMR at 75.4 MHz spectra, DMSO-d6, for compound **3b.**

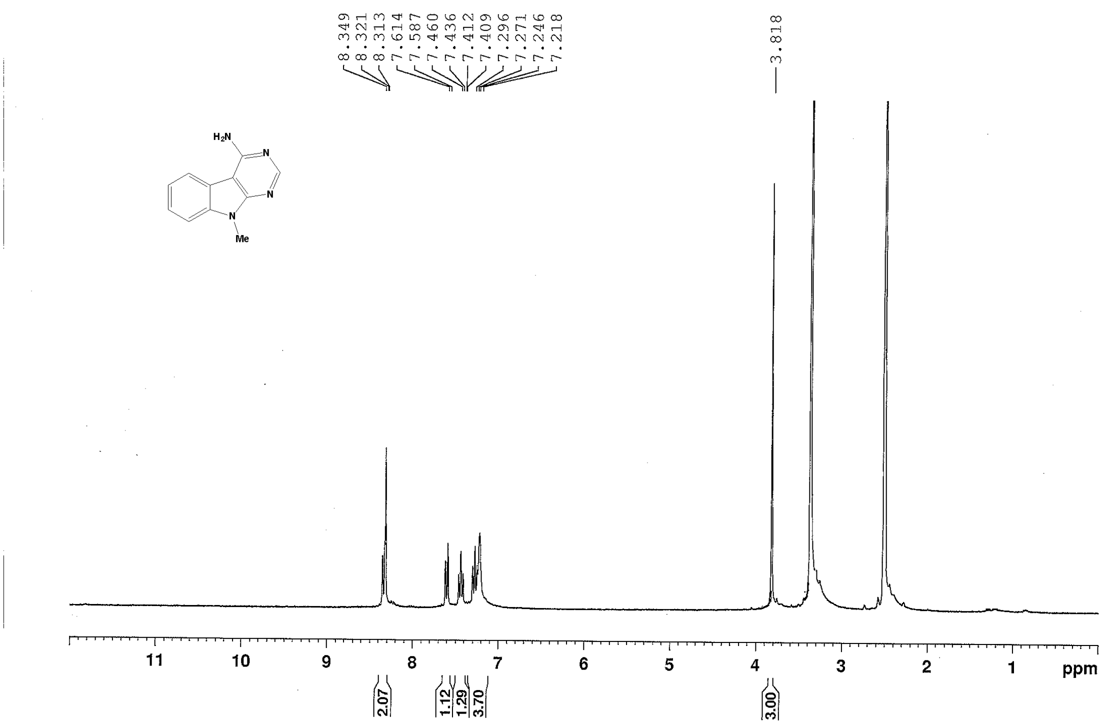

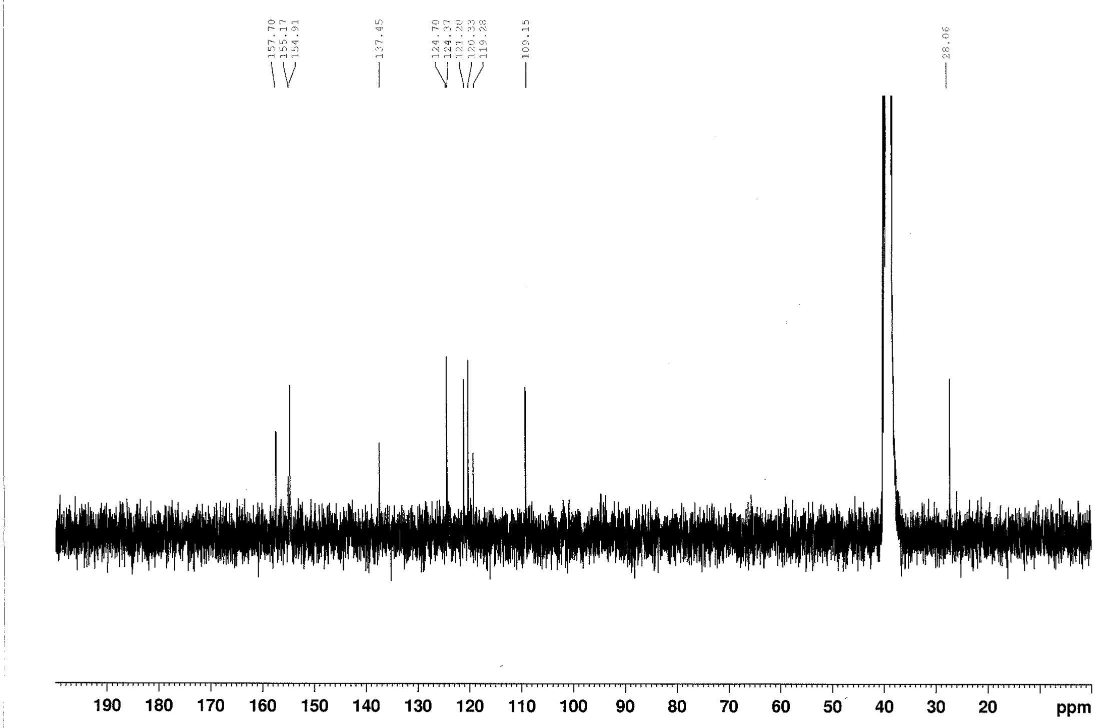

Figure S11. 1H NMR at 300 MHz and 13C NMR at 75.4 MHz spectra, DMSO-d6, for compound **3c.**

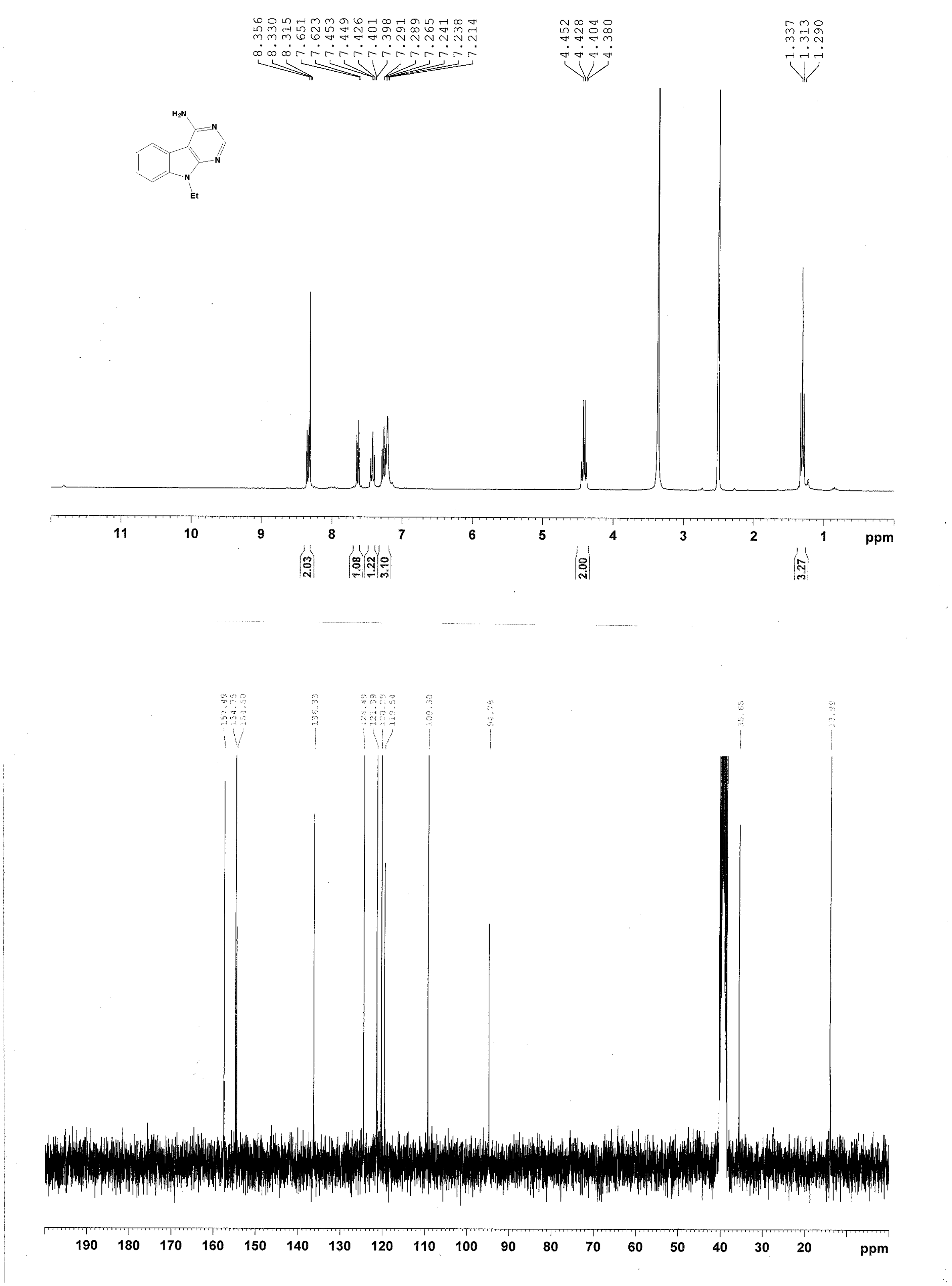

Figure S12. 1H NMR at 300 MHz and 13C NMR at 75.4 MHz spectra, DMSO-d6, for compound **3d.**

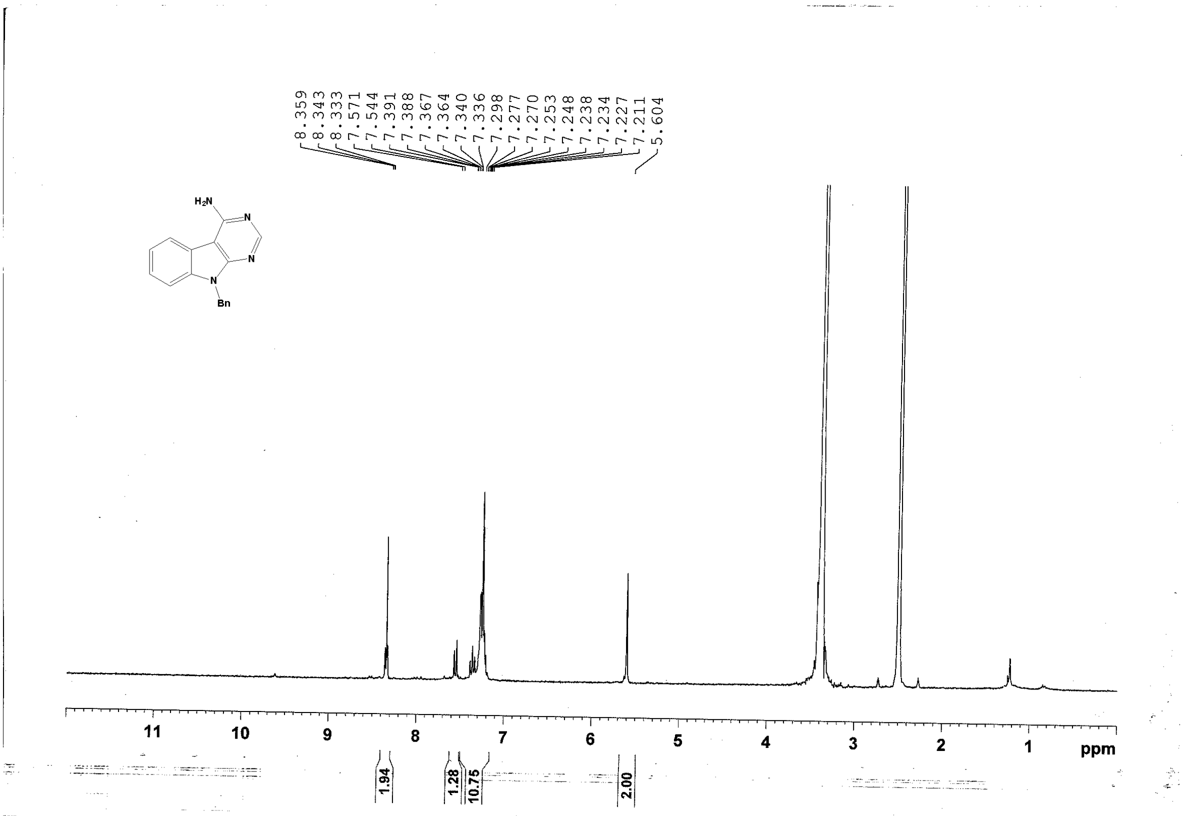

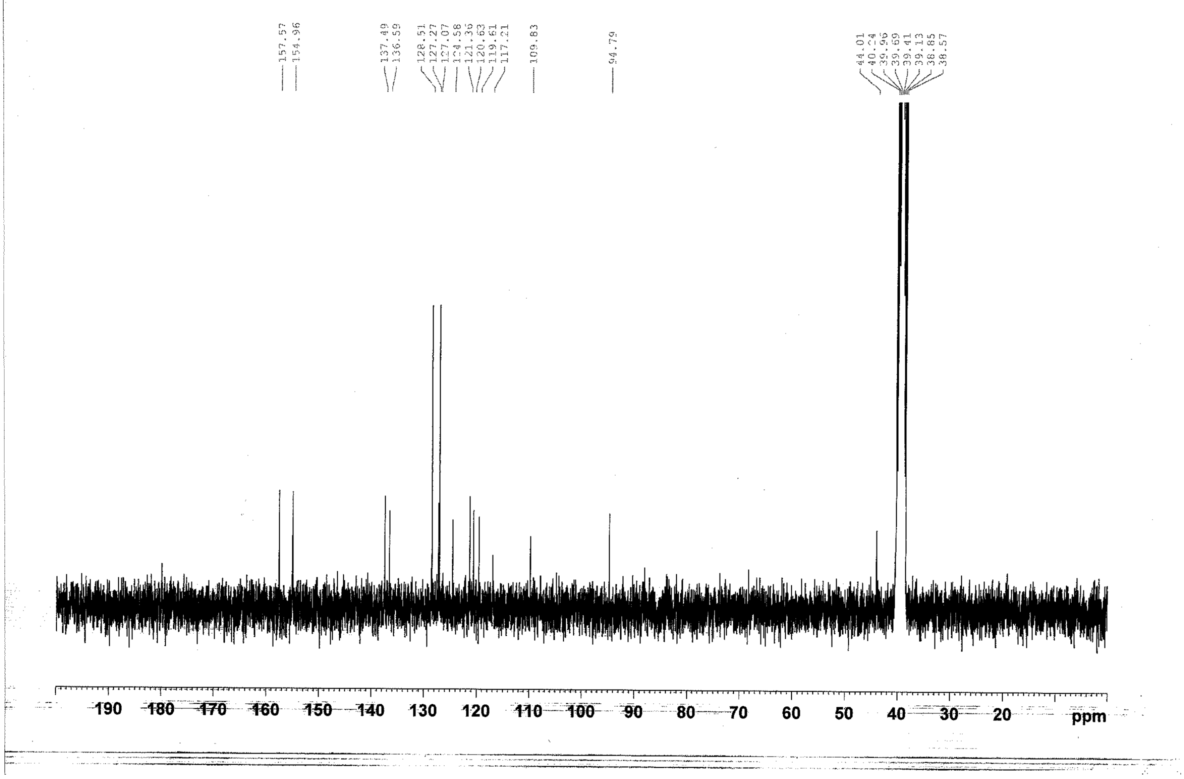

Figure S13. 1H NMR at 300 MHz and 13C NMR at 75.4 MHz spectra, DMSO-d6, for compound **4a.**

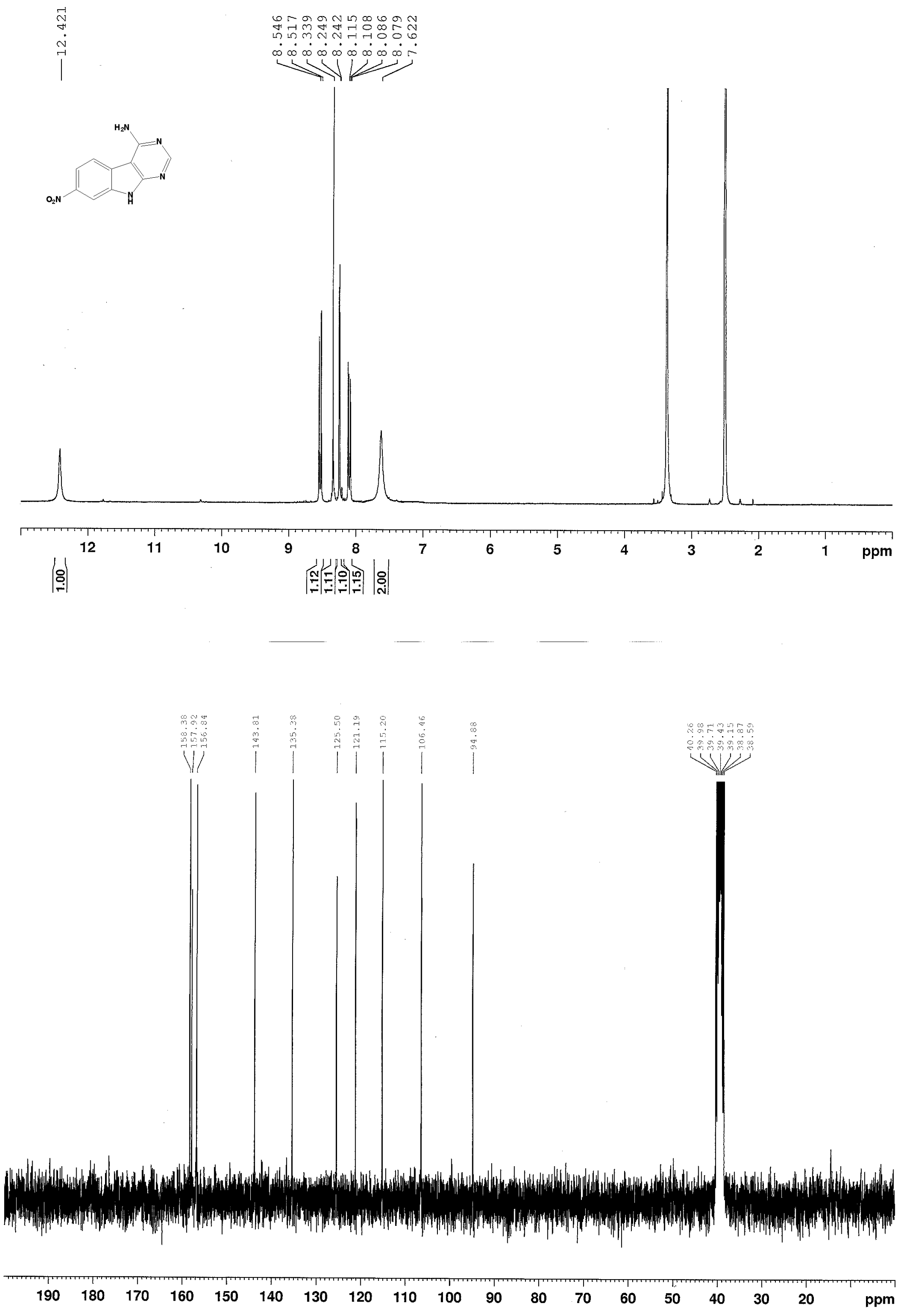

Figure S14 1H NMR at 300 MHz and 13C NMR at 75.4 MHz spectra, DMSO-d6, for compound **4b.**

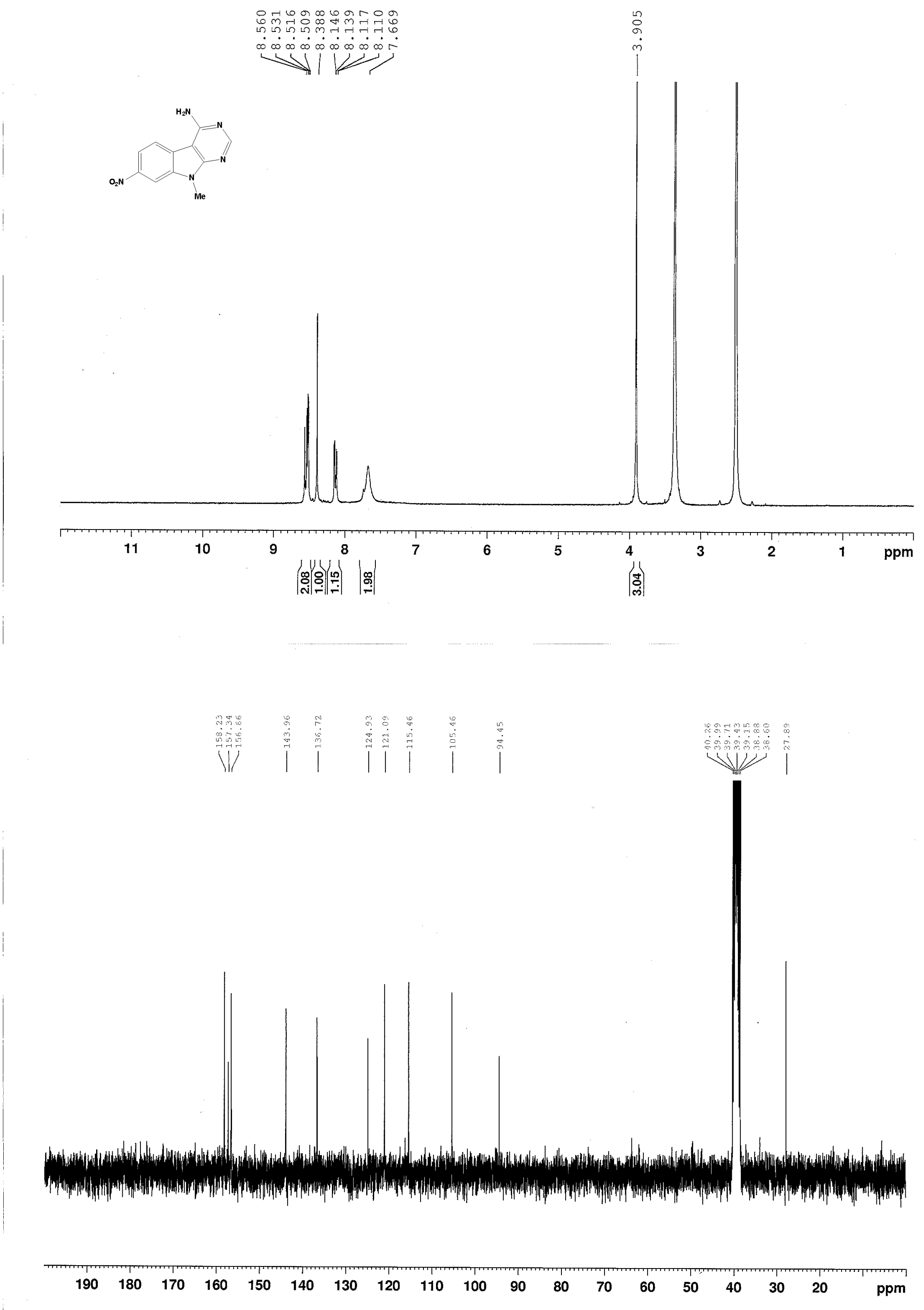

Figure S15 1H NMR at 300 MHz and 13C NMR at 75.4 MHz spectra, DMSO-d6, for compound **4c.**

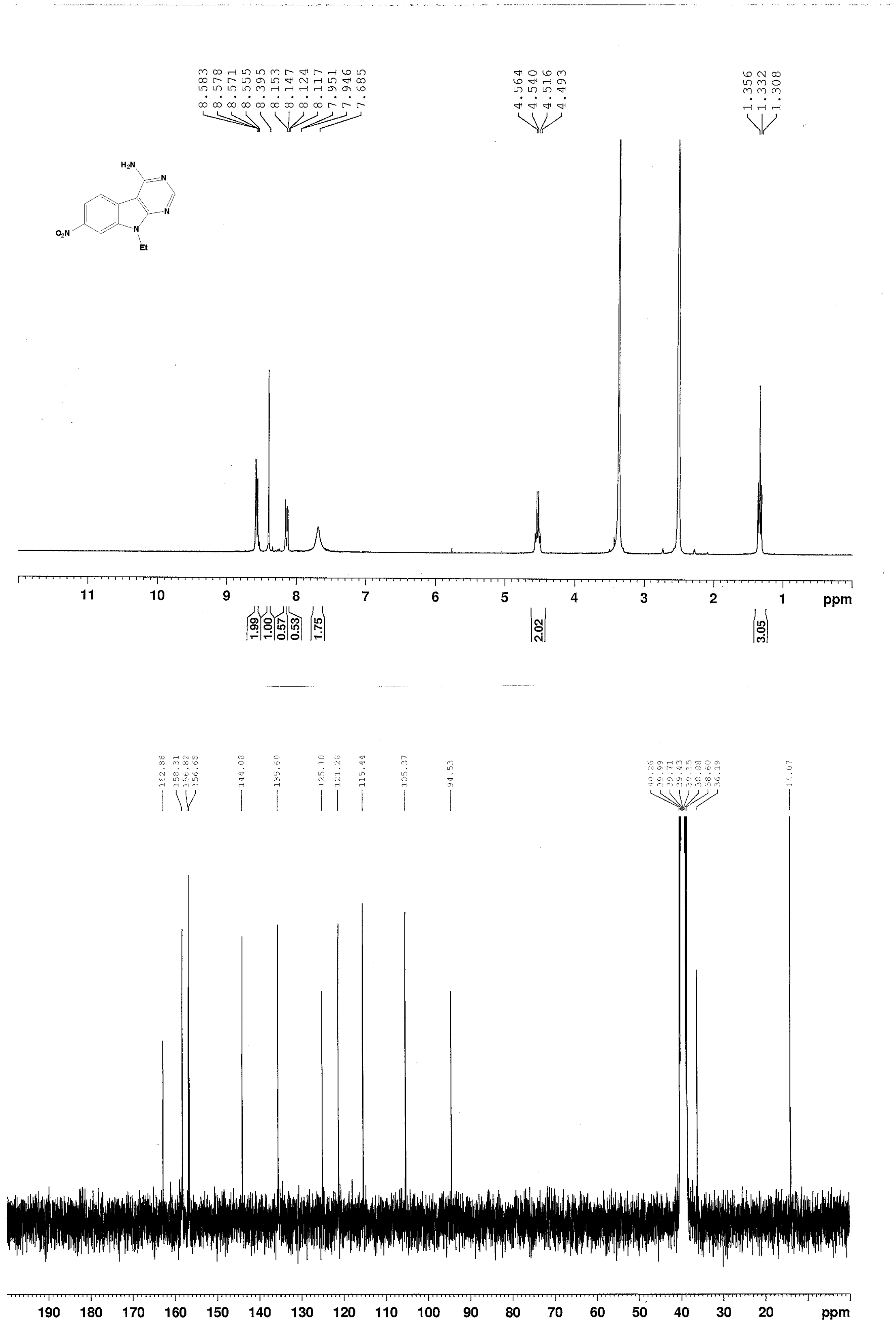

Figure S16 1H NMR at 300 MHz and 13C NMR at 75.4 MHz spectra, DMSO-d6, for compound **4d.**

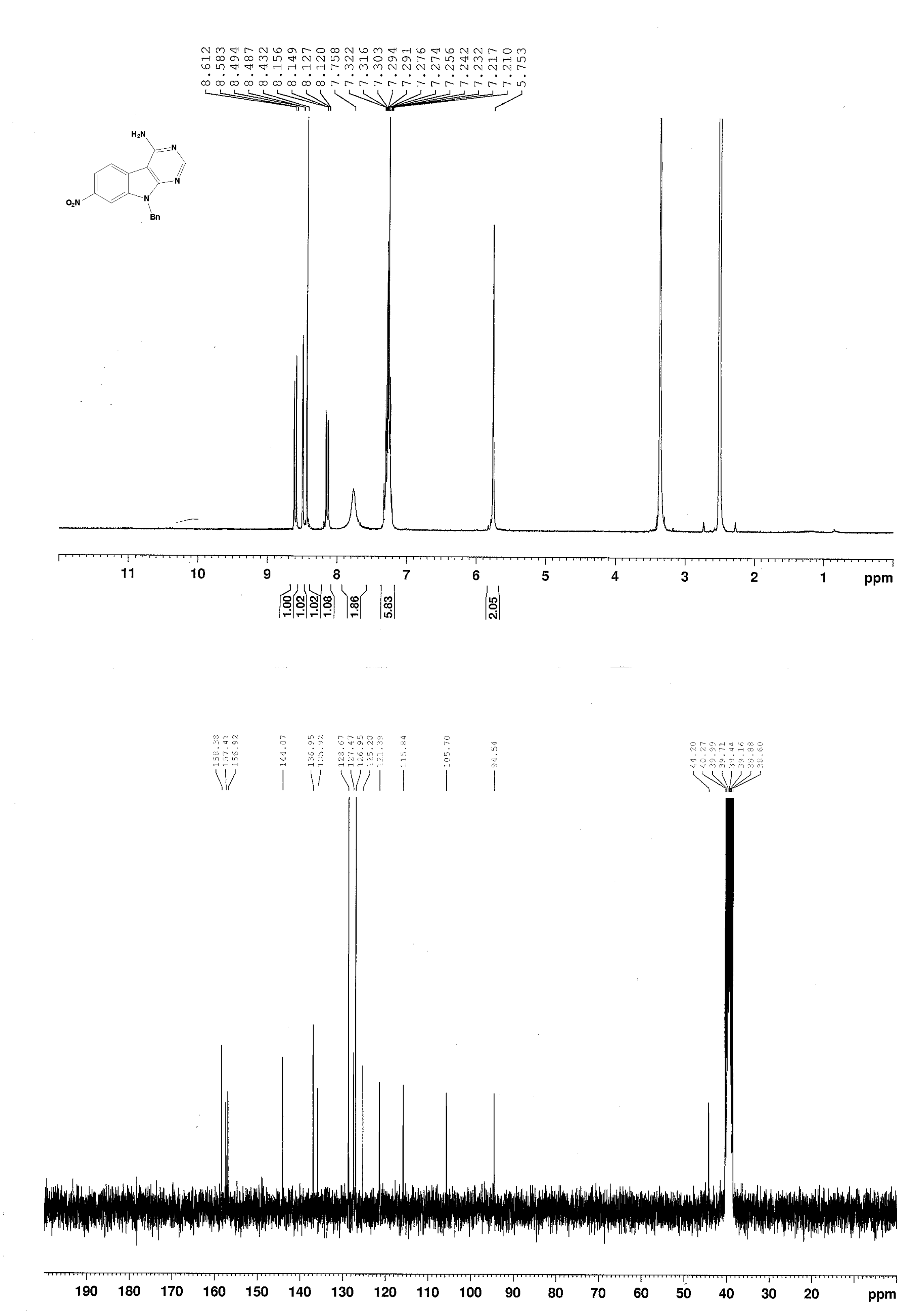

© 2020 by the authors. Licensee MDPI, Basel, Switzerland. This article is an open access article distributed under the terms and conditions of the Creative Commons Attribution (CC BY) license (http://creativecommons.org/licenses/by/4.0/).
